# Short-course combination treatment for experimental chronic Chagas disease

**DOI:** 10.1126/scitranslmed.adg8105

**Published:** 2023-12-13

**Authors:** Silvia González, Richard J. Wall, John Thomas, Stephanie Braillard, Gino Brunori, Isabel Camino Díaz, Juan Cantizani, Sandra Carvalho, Pablo Castañeda Casado, Eric Chatelain, Ignacio Cotillo, Jose M. Fiandor, Amanda Fortes Francisco, David Grimsditch, Martine Keenan, John M. Kelly, Albane Kessler, Chiara Luise, Jon J. Lyon, Lorna MacLean, Maria Marco, J. Julio Martin, Maria S. Martinez, Christy Paterson, Kevin D. Read, Angel Santos-Villarejo, Fabio Zuccotto, Susan Wyllie, Tim J. Miles, Manu De Rycker

**Affiliations:** 1 Global Health Medicines R&D, GSK, Tres Cantos, Madrid, Spain; 2 Global Investigative Safety, GSK, Ware, UK; 3 Discovery DMPK, GSK Tres Cantos, Madrid, Spain; 4 London School for Hygiene and Tropical Medicine, London, UK; 5 Wellcome Centre for Anti-Infectives Research, University of Dundee, Dundee, UK; 6 DNDi, Geneva, Switzerland; 7 Epichem, Bentley, Australia

## Abstract

Chagas disease, caused by the protozoan parasite *Trypanosoma cruzi*, affects millions of people in the Americas and across the world leading to considerable morbidity and mortality. Current treatment options, benznidazole (BNZ) and nifurtimox, offer limited efficacy and often lead to adverse side effects due to long treatment durations. Better treatment options are therefore urgently required. Here we describe a pyrrolopyrimidine series, identified through phenotypic screening, that offers a clear opportunity to improve on current treatments. *In vitro* cell-based washout assays demonstrate that compounds in the series are incapable of killing all parasites, however, combining these pyrrolopyrimidines with a sub-efficacious dose of BNZ can clear all parasites *in vitro* after five days. Importantly, these findings were replicated in a clinically predictive *in vivo* model of chronic Chagas disease, where five days treatment with the combination was sufficient to prevent parasite relapse. Comprehensive mechanism of action studies, supported by ligand-structure modelling, show that compounds from this pyrrolopyrimidine series inhibit the Q_i_ active site of *T. cruzi* cytochrome *b*, part of the cytochrome *bc1* complex of the electron transport chain. Knowledge of the molecular target enabled a cascade of assays to be assembled to evaluate selectivity over the human cytochrome *b* homologue. As a result, a highly selective and efficacious lead compound was identified. The combination of our lead compound with BNZ rapidly clears *T. cruzi* parasites, both *in vitro* and *in vivo*, and shows great potential to overcome key issues associated with currently available treatments.

## Introduction

Chagas disease, caused by the protozoan parasite *Trypanosoma cruzi*, is endemic in 21 Latin American countries, affecting an estimated seven million people and causing at least ten thousand deaths annually (1, 2). Chagas disease is thus one of the world’s largest parasitic killers after malaria. Approximately 30% of infected individuals develop chronic symptomatic disease that mainly affects the cardiovascular and digestive systems, leading to considerable morbidity and mortality in endemic countries (3). An estimated 1.2 million people are currently suffering from Chagas cardiomyopathy (4). Benznidazole (BNZ) and nifurtimox (NFX), the only drugs licensed for treatment of Chagas disease, suffer from multiple shortcomings including toxicity, prolonged treatment regimens (60 − 90 days), as well as limited efficacy in chronic patients (5–9). Discontinuation of treatment prior to completion is common (up to 29% for BNZ and 43% for NFX) due to adverse side-effects (10, 11). Preliminary clinical data with shortened BNZ regimens show lower toxicity while retaining efficacy, however larger clinical trials are required to confirm these findings, and variable efficacy is likely to remain an issue (12). Consequently, new treatment options with more favorable efficacy and toxicity profiles are urgently required (13), as described by the Target Product Profile for Chagas disease (14). Historically, drug discovery for neglected tropical diseases has been hampered by limited resources, which has encouraged drug repositioning as a rapid way to access novel treatments (15). Identified through repurposing, the antifungal drug posaconazole (POS), an inhibitor of C14α sterol demethylase (CYP51) critical for *T. cruzi* ergosterol biosynthesis (16), demonstrated *in vitro* antiproliferative activity against amastigotes and *in vivo* efficacy both in acute and chronic murine models of *T. cruzi* infection (17). However, when evaluated in a phase 2 clinical study, most patients relapsed one year after treatment (7). Similar results were obtained for fosravuconazole, another azole CYP51 inhibitor (18). Follow-up studies have demonstrated that CYP51 inhibitors are slow killers of parasites and are unable to eradicate all parasites either *in vitro* or in animal models of infection, perhaps offering an explanation for the clinical failure of these compounds (19–22). These findings highlight the need for detailed *in vitro* and *in vivo* profiling in appropriate models to identify compounds with a suitable mode of action for progression towards the clinic.

Recent studies have identified small subpopulations of *T. cruzi* that can survive prolonged drug treatment at high concentrations. While our understanding of these “persister” forms is in its infancy, there is no doubt they pose a major challenge to the development of antichagasic drugs. Indeed, *T. cruzi* persisters may contribute to the reduced efficacy of BNZ against chronic Chagas disease. Current data indicate that persister-related drug tolerance is transient and the result of reduced cell proliferation rates (19, 23). *In vitro* assays that incorporate a washout and outgrowth phase are being used increasingly to examine the ability of compounds to deliver sterile cure *in vitro* (19, 23–27) and may more accurately predict *in vivo* efficacy and human trial outcomes.

Tissue and organ damage observed in Chagas disease patients is caused by the parasite and the host’s inflammatory immune response to the infection (6, 28). *T. cruzi* is capable of parasitizing any nucleated cell, with parasites detected in cardiac and skeletal muscle, smooth muscle myocytes, bladder, connective tissues, enteric nerves and adipocytes as well as other tissues in infected animals (29–31). In humans, parasites have been detected in cardiac tissues of Chagas disease patients with inflammatory cardiomyopathy, in adipose tissue, in smooth muscle and in the GI tract (32–35). This wide tissue tropism poses a second major challenge for Chagas disease drug discovery since a successful drug will need to reach all parasite reservoirs at sufficiently high concentrations to eliminate all parasites.

Here, we sought to develop a treatment for Chagas disease capable of overcoming the deficiencies associated with current therapeutics. Our work also explored the value of *in vitro* washout assays with respect to outcomes in an animal model of chronic Chagas disease. A promising pyrrolopyrimidine series was identified from a phenotypic screen with detailed mechanism of action studies identifying *T. cruzi* cytochrome *b* as the molecular target. Target identification enabled subsequent target-focused development of a highly selective advanced lead compound.

## Results

### Identification and *in vitro* characterization of pyrrolopyrimidine series

**Fig. 1:**
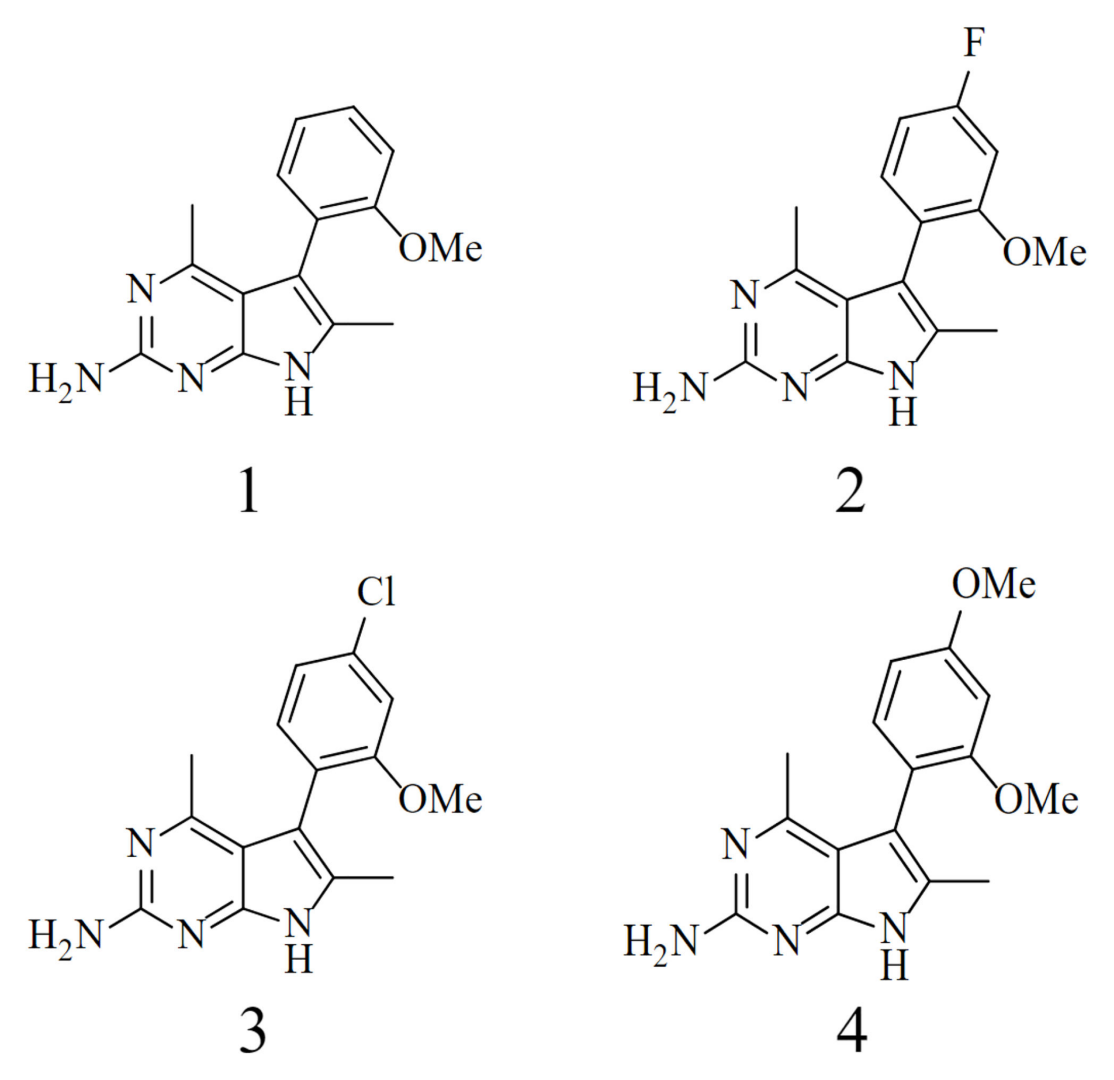
Compounds. Structures of key compounds in the pyrrolopyrimidine series. All compounds have 4,6-dimethyl-7H-pyrrolo[2,3-d]pyrimidine as core scaffold. Compound 1: 5-(2-methoxyphenyl)-4,6-dimethyl-7H-pyrrolo[2,3-d]pyrimidin-2-amine, starting point of the series, identified through library screening. Compound 2: 5-(4-Fluoro-2-methoxyphenyl)-4,6-dimethyl-7H-pyrrolo[2,3-d]pyrimidin-2-amine. Compound 3: 5-(4-Chloro-2-methoxyphenyl)-4,6-dimethyl-7H-pyrrolo[2,3-d]pyrimidin-2-amine. Compound 4: 5-(2,4-Dimethoxyphenyl)-4,6-dimethyl-7H-pyrrolo[2,3-d]pyrimidin-2-amine. Reaction schemes are shown in Fig. S1.

A cell-based high-throughput screen of GSK’s compound collection was carried out against the intracellular amastigote form of *T. cruzi* (36). The progression criteria of DNDi’s (Drugs for Neglected Diseases *initiative*) (13, 20) were used as a guide to assess screening hits and led to the prioritization of compound 1 (Fig. 1, Table 1, Fig. S1). Upon resynthesis, the 4,6-dimethyl-7*H*-pyrrolo[2,3-*d*]pyrimidine derivative was confirmed as moderately potent against intracellular amastigotes (pEC_50_ of 6.5) with no apparent cytotoxicity against either Vero host cells or uninfected HepG2 cells (pEC_50_ <4.5). In addition, a direct biochemical assay with recombinant *T. cruzi* CYP51 demonstrated that compound 1 did not inhibit this enzyme, confirming the pyrrolopyrimidine series exploits a different mode of action to the clinically invalidated azoles posaconazole and fosravuconazole. These promising preliminary findings along with the optimal lipophilicity and chemical tractability of the pyrrolopyrimdine scaffold (Table 1) prompted an early-stage drug discovery program aimed at identifying a preclinical candidate for Chagas disease.

Structural variations of compound 1 were synthesized to characterize the structure-activity relationships within this series. These studies revealed the importance of the 4,6-dimethyl-7*H*-pyrrolo[2,3-*d*]pyrimidin-2-amine core for *T. cruzi* activity, and ultimately led to the development of compound 2 (Fig. 1, Table 1, Fig. S1). Compared with the original screening hit, this analogue demonstrated improved activity against *T. cruzi*, with similar potency against amastigotes and trypomastigotes. Activity against the non-dividing trypomastigotes, in combination with the lack of inhibition of *Tc*CYP51, confirms the series has a different mechanism of action compared to that of posaconazole and similar azoles, as CYP51 inhibitors are inactive against this life-cycle stage (Table 1)(19). Assessment of the physicochemical properties of compound 2 revealed an acceptable Property Forecast Index (PFI) (37), that correlates with high solubility in both aqueous media and fasted-state simulated intestinal fluid (FaSSIF) (Table 1). Compound 2 displayed an optimal *in vitro* ADME profile, with high metabolic stability in mouse and human microsomes and low binding to plasma proteins in both species, thus warranting further *in vivo* studies in a murine model of *T. cruzi* infection (Table 1).

### *In vivo* characterization of the pyrrolopyrimidine series

Compound 2 was evaluated in a bioluminescent mouse model of Chagas disease designed to mimic chronic infections. This model successfully differentiates the clinically used drugs from the clinically invalidated CYP51 inhibitors (21). Key features of this model include immunosuppression of mice following cessation of treatment to allow detection of parasite relapses, and *ex vivo* bioluminescence imaging that allows tissue-specific visualization of parasites. Treatments are considered efficacious if there is no evidence of bioluminescence during *in vivo* (mouse) and *ex vivo* (organ/tissue) imaging following immunosuppression (38). Under these experimental conditions, compound 2 was able to prevent parasite relapse in 2 of the 6 mice treated orally twice daily for 10 days at 50 mg/kg (Table S1). Increasing the dose to 100 mg/kg or extending treatment duration to 21 days provided similar partial efficacy rates. Compound 3, which showed enhanced potency against *T. cruzi* while maintaining a suitable physicochemical profile (Table 1), also failed to achieve full efficacy (Table S1).

Bioanalysis of blood samples obtained from infected, orally dosed mice in each study (Table S2) indicated that free (protein unbound) compound levels in blood remained above the *in vitro* EC_50_ for the duration of dosing. Although the series displayed potency against infective (trypomastigote) and replicative (amastigote) parasite stages and exhibited high free blood exposure, it was unable to confer full efficacy in the murine model employed. To gain insights into the lack of *in vitro* to *in vivo* translation, we next focused on exploring potential drivers for this disconnection.

### Failure to clear all parasites *in vitro*

**Fig. 2:**
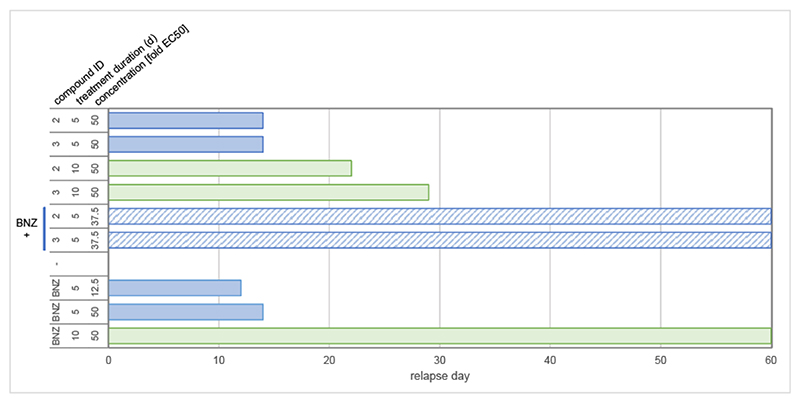
Washout outgrowth assay. Intracellular parasites were subjected to treatment with compounds 2, 3 and BNZ at the indicated concentrations and for either five days (blue) or 10 days (green), followed by extensive washout of compounds and assessment of parasite relapse for 60 days post washout. Relapse day indicates the first day after washout that viable parasites were observed microscopically. Combination treatments (hashed bars) used BNZ at 12.5-fold EC_50_.

Since different *T. cruzi* strains were used in our *in vitro* (Silvio X10/7 A1) and *in vivo* (CL Brener) assays, we hypothesized that this variation may play a role in the discrepancy between *in vitro* potency and *in vivo* efficacy. Several compounds from the series were profiled against a panel of strains, including the strain used in the animal model. The compounds evaluated demonstrated similar or improved potency against these strains compared to Silvio X10/7 A1 (Fig. S3). We next investigated the *in vitro* rate-of-kill (39) achieved by compounds from the pyrrolopyrimidine series and found that they are able to kill intracellular amastigotes, with rapid onset of action (Fig. S4). Since the above *in vitro* assays cannot distinguish compounds that reduce parasite levels from those capable of sterilizing, it is possible that a small sub-population of parasites survive treatment with the pyrrolopyrimidines. *T. cruzi* persister parasites have been previously described (19, 23, 40), and incomplete killing of the parasite population could explain the partial relapses seen *in vivo*. Washout outgrowth assays were performed to detect the presence of viable and growth competent parasites after treatment (Fig. 2). *T. cruzi* cultures exposed to compounds 2, 3 or BNZ at concentrations equivalent to 50× their respective EC_50_ values for five days all relapsed 14 days after the end of treatment. Ten-day treatment with compounds 2 and 3 also resulted in relapses between 22 and 29 days after compound washout. Under these conditions no relapse was seen with BNZ-treated cultures. Further extension of treatment duration to 16 days still resulted in relapse (Fig. S5). The recrudescing parasites in this last experiment retained sensitivity to compound 2, indicating that we did not select for genetic resistance in these washout experiments. These results indicate that, while these pyrrolopyrimidines are cidal, they are unable to clear all parasites *in vitro* even following extensive treatments which provides a likely explanation for the relapses observed in our *in vivo* experiments.

### Short course combination treatment prevents relapse

Our aim is to develop new Chagas disease drugs that can provide sterile cure for patients. Based on the results above, the series presented here is unlikely to be able to achieve this goal alone. Therefore, we sought to explore the potential for these pyrrolopyrimidines to be used as a combination treatment with BNZ to achieve maximum efficacy using a short dosing regimen. We used our washout outgrowth assay to assess the potential of BNZ-pyrrolopyrimidine combinations in a 5-day regimen to effect sterile cure *in vitro*. Strikingly, when an ineffective concentration of BNZ (12.5-fold EC_50_) was combined with pyrrolopyrimidine series compounds at 37.5-fold EC_50_, no relapse was observed after just five days of treatment (Fig. 2). The total drug burden in these experiments was 50-fold EC_50_, based on an additive interaction between BNZ and the pyrrolopyrimidine series (Fig. S6) (41) allowing direct comparison with our previous monotherapy experiments. A similar experiment comparing the effectiveness of posaconazole and compound 2 as combination partners for BNZ (Fig. S7) showed that, in contrast to the pyrrolopyrimidine − BNZ treatment, combination with the azole failed to eliminate all parasites, despite a longer treatment duration (8-days) and a higher combined dose, relative to the experiment in Fig. 2.

**Fig. 3:**
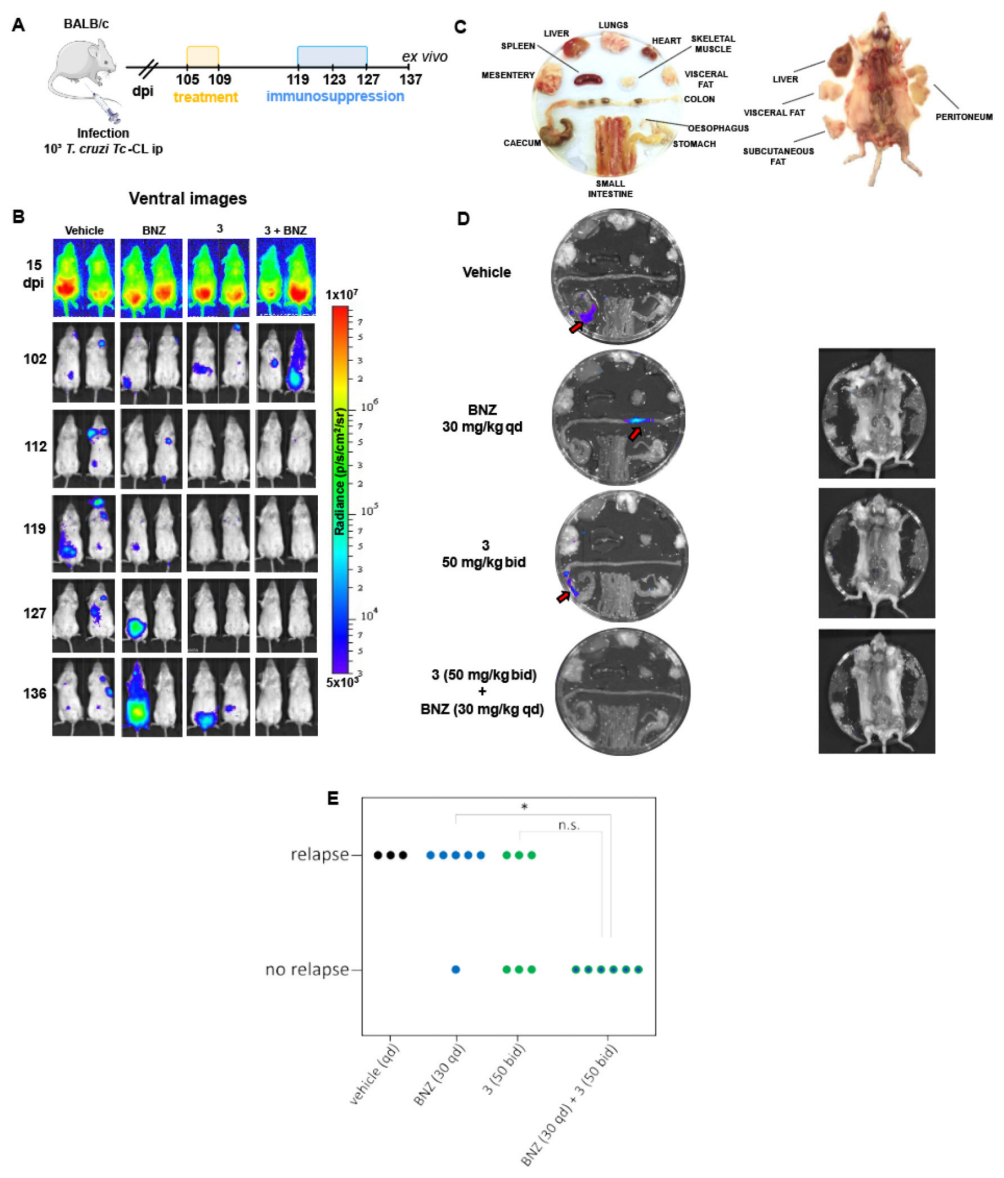
*In vivo* assessment of the combination of compound 3 with a low dose of BNZ in a murine model of chronic *T. cruzi* infection. (A) Schematic representation of experiment. Parts of the figure were drawn by using pictures from Servier Medical Art. Servier Medical Art by Servier is licensed under a Creative Commons Attribution 3.0 Unported License (https://creativecommons.org/licenses/by/3.0/).” Bioanalysis data for this experiment is shown in Table S3. (B) Representative ventral images of BALB/c mice at the chronic stage of infection (105 days) treated orally for 5 days with vehicle (once daily (qd), n = 3), BNZ (30 mg/kg, qd, n = 6), compound 3 (50mg/kg, twice daily (bid), n = 6), BNZ (30 mg/kg, qd) in association with compound 3 (50mg/kg, bid, n=6). Bioluminescent negative mice were immunosuppressed on days 119, 123 and 127 using cyclophosphamide (200 mg/kg i.p.). For welfare reasons, we did not continue immunosuppression if there were definitive indications of a relapse. Heat-maps are on log10 scales and indicate intensity of bioluminescence from low (blue) to high (red). (C) *Ex vivo* dissection and imaging guide (D) At day 137 post-infection organs were removed and assessed for *ex vivo* bioluminescence. Representative *ex vivo* images of organs (left) and carcass (right) are shown. Red arrows indicate infection foci. None of the mice treated with combination therapy showed signs of relapse. (E) Outcomes plot: mice were designated as not relapsed only when bioluminescent-negative in both in vivo and *ex vivo* imaging. Outcome for each animal plotted for each treatment. Fisher’s exact test was used to calculate statistical significance (α = 0.05, * = significant, n.s. = non-significant). 95% confidence intervals for no-relapse were calculated with the Adjusted Wald method (85) and are as follows: BNZ (30 qd) [0.01 − 0.58], 3 (50 bid) [0.19 − 0.81] and BNZ + 3 [0.64 − 1]. Treatment colors: vehicle = black, BNZ = blue, cpd 3 monotherapy = green, BNZ + cpd 3 combination = blue/green. Treatment regimens on y axis are for 5 days and expressed as mg/kg).

Encouraged by the apparent sterilizing effect seen in the washout assay, we proceeded to test the combination potential of compound 3 with BNZ in our chronic Chagas disease animal model. Since one of the shortcomings of BNZ is cumulative toxicity (42), lowering the dose and/or shortening the length of treatment could constitute a marked improvement to current treatment regimens. Thus, a suboptimal oral dose of BNZ (30 mg/kg qd) was combined with a 50 mg/kg bid dose of compound 3 in a 5-day dosing regimen. In line with the *in vitro* washout data, this approach prevented relapse in all six treated animals (Fig. 3A-E), while three out of six animals treated with compound 3 monotherapy relapsed. Although this difference was not statistically significant within this individual experiment (Fig. 3E), across multiple experiments with dosing regimens of up to 21 days, there were always relapses with monotherapy treatments (data for compounds 2, 3 and 4 in Table S1, Fig. 3 and Fig. 6), indicating that the lack of relapses with the short combination treatment is likely meaningful. In view of this promising efficacy data, and the close alignment of the *in vitro* washout experiments with the *in vivo* efficacy studies we decided to pursue the development of the pyrrrolopyrimidine − BNZ co-administration approach.

Our *in vitro* washout and *in vivo* efficacy results indicate a level of cooperation between BNZ and the pyrrolopyrimidine series that results in rapid clearance of all parasites including persisters. To understand if this combination provided an advantage over the monotherapies in terms of rate-of-kill we carried out *in vitro* combination rate-of-kill experiments. Interestingly, the fast rate-of-kill observed with BNZ at 50 μM (Fig. S8) can be replicated with as little as 10 μM BNZ when combined with 0.02 μM compound 3. Moreover, the fast rate-of-kill observed for BNZ at 50 μM can be further accelerated by combining with compound 3. These results are in line with the rapid clearance of parasites observed in the *in vivo* studies.

### Elucidation of mode of action Cross-resistance studies

**Fig. 4:**
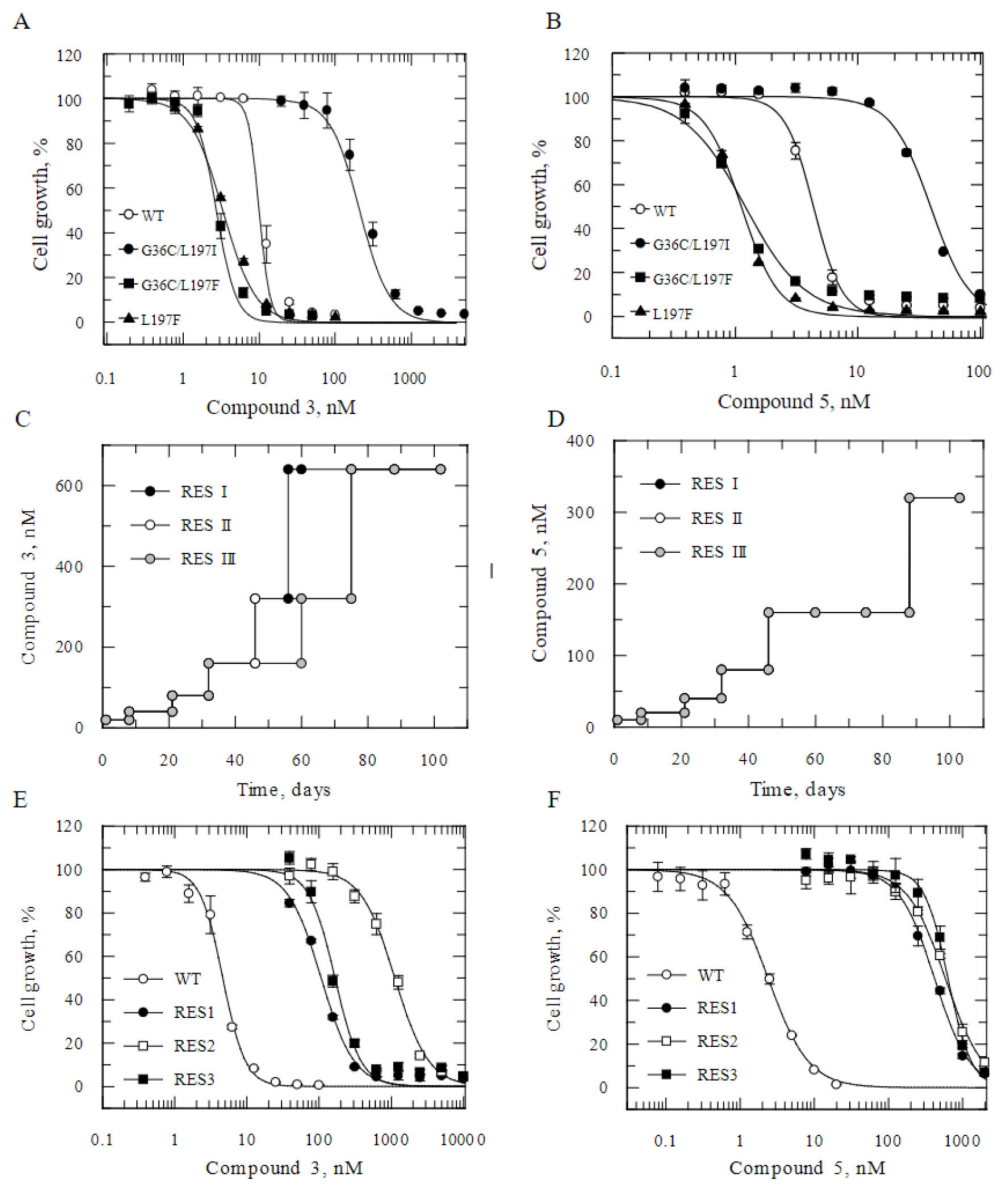
Resistance profiling of compound 3 and compound 5. Cross-resistance profiling: EC_50_ values were determined for compound 3 (A) and compound 5 (B) against cell lines bearing mutations in cytochrome *b* (*cytb*). For compound 3, wild-type epimastigotes (open circles) returned an EC_50_ value of 11.3 ± 0.5 nM, while values of 258 ± 7, 3.0 ± 0.1 and 3.7 ± 0.1 nM were determined for cell lines bearing G36C/L197I (closed circles), G36C/L197F (closed squares) and L197F (closed triangles) mutations in *cytb*, respectively. For compound 5, wild-type epimastigotes returned an EC_50_ value of 11.3 ± 0.5 nM, while values of 37 ± 2, 1.2 ± 0.1 and 1.1 ± 0.1 nM were determined for cell lines bearing G36C/L197I, G36C/L197F and L197F mutations in *cytb*, respectively. Representative dose-response curves shown. (C-F): Resistant line generation *in vitro*. (C and D): Schematic representation of the generation of compound 3 and compound 5 resistant lines in *T. cruzi* epimastigotes, respectively. Resistant cell lines 1 − 3 (black, white, grey, respectively) are shown for each compound (for compound 5 all three cell lines overlap completely). X-axis shows drug concentration that cells are grown in. (E) EC_50_ values for compound 3 were determined for wild-type (open squares, 4.6 ± 0.2 nM) and the three resistant clones for each compound (RES1: solid circles, 107 ± 5.4 nM; RES2: open squares, 1120 ± 49 nM; RES3: closed squares, 166 ± 14 nM). (F) EC_50_ values for compound 5 were determined for wild-type (open squares, 2.4 ± 0.1 nM) and the three resistant clones for each compound (RES1: solid circle, 417 ± 11 nM; RES2: open squares, 585 ± 30 nM; RES3: closed squares, 635 ± 33 nM). Representative dose-response curves shown.

Following the promising combination efficacy results, we investigated the mode of action of the pyrrolopyrimidine series. As a first step, two representative compounds (compounds 3 and 5; Figs. 1 and S2) were screened against our panel of cell lines resistant to compounds with defined molecular targets. *T. cruzi* epimastigotes with two specific mutations (G36C and L197I) in cytochrome *b* (43), a key component of complex III of the electron transport chain (ETC) (44), demonstrated considerable cross-resistance to both compound 3 (23-fold, Fig. 4A) and compound 5 (9-fold, Fig. 4B). In contrast, epimastigotes bearing coincident G36C and L197F mutations in cytochrome *b*, as well as parasites with a single L197F mutation, were hypersensitive to both compounds. It should be noted that both crossresistant and hypersensitive cell lines were originally evolved through *in vitro* exposure to specific inhibitors of the ubiquinone reduction center of cytochrome *b* (Qi site) (43). Indeed, both inhibitors used to generate resistance are known to exploit the same binding pose as the archetypal Qi site inhibitor antimycin A, and their resistance-conferring mutations cluster in close proximity to this active site.

### Resistance generation and whole genome sequencing

Cell lines resistant to compounds 3 and compound 5 were generated by exposing three independent cultures of clonal, drug-susceptible epimastigotes to increasing concentrations of compound *in vitro* (Fig. 4C and D). At the point where compound-exposed parasites could grow at concentrations equivalent to ~30× the established EC_50_ values of both compounds, resistant parasites were cloned by limited dilution and their susceptibility to compound 3 and compound 5 determined (Fig. 4E and F). Resistant clones selected with compound 3 were between 23 and 243-fold less sensitive than the wild-type parental cell line, while clones selected with compound 5 were between 173 and 265-fold less sensitive. Importantly, clones selected for resistance to compound 3 and compound 5 were reciprocally cross-resistant and cross-resistant to all the analogues within this series tested (Table 2). Collectively, these data indicate that all the compounds within this series are likely to share the same mechanism of action.

Whole genome sequencing (WGS) revealed that all six compound 3- and compound 5-resistant-clones maintained mutations within the gene encoding cytochrome *b* (*cytb*) (Table S4). No other homozygous mutations, or mutations in common between clones, were identified. Two of the compound 3-resistant clones shared the same L197I mutation, while the remaining four clones bore a F222L mutation. Parasites bearing the F222L mutation demonstrated far higher levels of resistance to compounds from within this chemical series than those with the L197I mutation. Of note, both mutations mapped to the Qi site. *Cytb* is encoded solely by the maxi-circle DNA, a minor component of the parasite’s kinetoplast DNA (45), equivalent to mitochondrial DNA in mammalian cells. These concatenated networks of mitochondrial DNA can maintain up to 50 copies of maxicircle DNA and thus up to 50 copies of *cytb*. This and other factors effectively preclude investigating the role of specific *cytb* mutations in resistance by standard genetic methods. Nevertheless, these data strongly suggest that mutations within *cytb* are driving resistance to compounds from this compound series.

### Compounds from the pyrrolopyrimidine series inhibit complex III of the ETC

To provide direct evidence that compounds from this series inhibit complex III, *T. cruzi* epimastigote clarified cell lysates enriched in mitochondria were prepared. Complex III activity within these lysates was monitored in the presence and absence of compound 3, compound 5 and related analogues from this series, using decylubiquinol as a pseudo-substrate (43). As expected, all compounds tested from this series proved to be potent inhibitors of complex III activity with IC_50_ values ranging from 1-8 nM (Table 2). These values correlate well with the EC_50_ values returned for these compounds against *T. cruzi* epimastigotes and support our hypothesis that cytochrome *b* is the principal molecular target of this series.

### Characterization of binding site through modelling

**Fig. 5:**
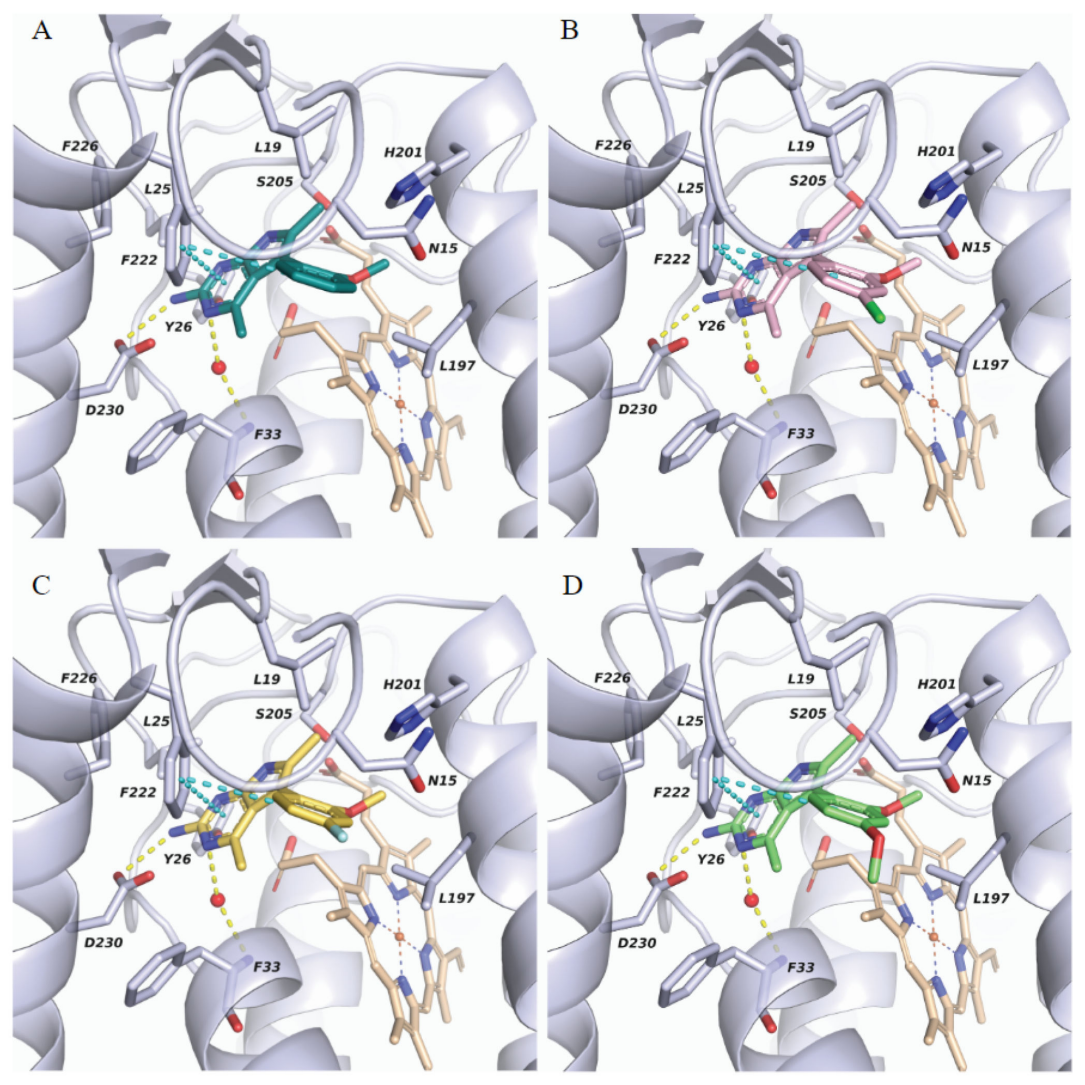
Predicted binding modes of compounds 1-4 at the *T. cruzi* cytochrome *b* pocket. (A) Compound 1. (B) Compound 3. (C) Compound 2. (D) Compound 4. For clarity only the side chains of the surrounding amino acid residues are shown, as light grey sticks; for residue F33, the backbone atoms are also shown. The heme group is depicted as beige sticks, while the oxygen atom of the conserved water molecule as a red sphere. Binding interactions are represented with dashed lines colored in yellow (hydrogen bond) and cyan (π-π stacking).

To gain insight into the binding mode of our compounds at the *T. cruzi* cytochrome *b* pocket and to rationalize the role of L197I and F222L mutations, we carried out molecular modelling studies. Firstly, compounds 1−4 were docked into a previously generated homology model of *T. cruzi* cytochrome *b* (43). These docking studies suggested that all compounds share a similar binding mode in which the 2-aminopyrimidine moiety establishes hydrogen bonds with D230 and a conserved water molecule interacting with the backbone nitrogen of F33 (Fig. 5 and Fig. S9). Further interactions, namely π-π stacking between F222 and both the pyrimidine and phenyl rings in addition to hydrophobic contacts with L19 and L197, contribute to stabilizing the inhibitors’ binding into the pocket. Interestingly, the 2-aminopyrimidine moiety adopts the same position as the antimycin headgroup in the Qi site of the bc1 complex (PDB ID: 1PPJ, (46)) and mimics the π-π stacking and two of the hydrogen bond interactions observed in the crystal structure (Fig. S10).

Molecular dynamics (MD) was used to further explore the binding hypothesis generated by docking. In particular, the best scoring solution for compound 3 bound to cytochrome *b* was simulated by MD for 100 ns. The analysis of the MD trajectory indicated that the binding mode is stable, and the interactions are preserved throughout the simulation. It is worth noting that fluctuations of the 4-chloro-2-methoxyphenyl moiety are detected affecting the π-π stacking of the phenyl ring with F222 (occupancy rate 8%). In contrast, the position of the heterocyclic core is stable, and the interactions formed by the 2-aminopyrimidine moiety are well conserved. The ligand-protein interaction diagram with the interaction occurrences and the ligand root-mean-square fluctuations (RMSF) are reported in Fig. S11 with the root-mean-square deviation (RMSD), the binding mode at different MD time steps and the protein-ligand contacts shown in Fig. S12 and Fig. S13.

After validating the docking hypothesis through MD simulation, we investigated the impact of the L197I and F222L mutations on ligand binding. Based on our docking results, both L197 and F222 residues are in direct contact with the ligand (Fig. S14) hence their mutation is likely to impact the ligand recognition event. Specifically, F222 contributes to defining the shape of the pocket and establishes π-π stacking and hydrophobic contacts with the compounds. Mutation of F222 to leucine changes the morphology of the binding site and removes specific interactions critical for ligand binding. Similarly, L197 forms hydrophobic contacts with the ligands but the mutation to the homologous isoleucine probably has a minor effect on pocket shape and ligand interaction. Nevertheless, to corroborate this assumption, docking studies were performed in the mutated binding sites. As expected, the impact of L197I is slight, and the same binding mode as in the wild-type (WT) was retrieved (Fig. S15 and Fig. S16). Less extended hydrophobic contacts with I197 compared to L197 are present, in keeping with the relatively modest levels of resistance demonstrated by parasites with this specific mutation (Table 2). Docking studies demonstrate how the F222L mutation completely disrupts the ligand binding mode, highlighting the critical role of this residue in the ligand and the reason that parasites bearing the F222L mutation demonstrated such high levels of resistance to these compounds. As an example, the three best scoring docking poses obtained for compound 3 in the F222L mutant are reported in Fig. S17 and they are all different from the binding pose in the WT.

### Identification of a selective *T. cruzi* bc1 inhibitor

Complex III of the mitochondrial electron transport chain is conserved across eukaryotic species. Therefore, testing compounds to prioritize those selectively inhibiting the *T. cruzi* enzyme is warranted as off-target inhibition of the mammalian cytochrome *b* and *bc1* complex has precedent for undesirable mitochondrial toxicities preventing clinical development (47). Compound 3 did not inhibit human complex III at concentrations up to and including 66.7 μM, however a 20% reduction in activity was observed at 200 μM (Table 3). To evaluate interference with overall mitochondrial function, not limited to electron transport chain inhibition, additional mitochondrial functional assays were performed. Compound 3 reduced the calcium loading capacity (CLC) of isolated rat liver mitochondria in a concentration dependent manner, with a minimum effective concentration (MEC) of 5.7 μM (Table 3). Mitochondrial dysfunction was further corroborated with the Seahorse MST (Mitochondrial Stress Test) assay in HepG2 cells, where the oxygen consumption rate (OCR) was reduced (>15%) at 80 μM.

The established CLC assay was used as a counter screen to identify compounds within the pyrrolopyrimidine series with less potential for mitochondrial toxicity. Compound 4 was identified as a selective *T. cruzi* complex III inhibitor, with reduced impact on both human complex III and isolated rat liver mitochondria (CLC assay) (Table 3). Further evaluation in the Seahorse MST confirmed less interference with mitochondrial activity in HepG2. Compound 4 showed the same promising combination efficacy as compounds 2 and 3 in the *in vitro* washout assay (Fig. S18) and exhibited an overall favorable physicochemical profile (Table 1). Based on this and the mitochondrial selectivity profile compound 4 was progressed to our murine model of chronic *T. cruzi* infection.

**Fig. 6:**
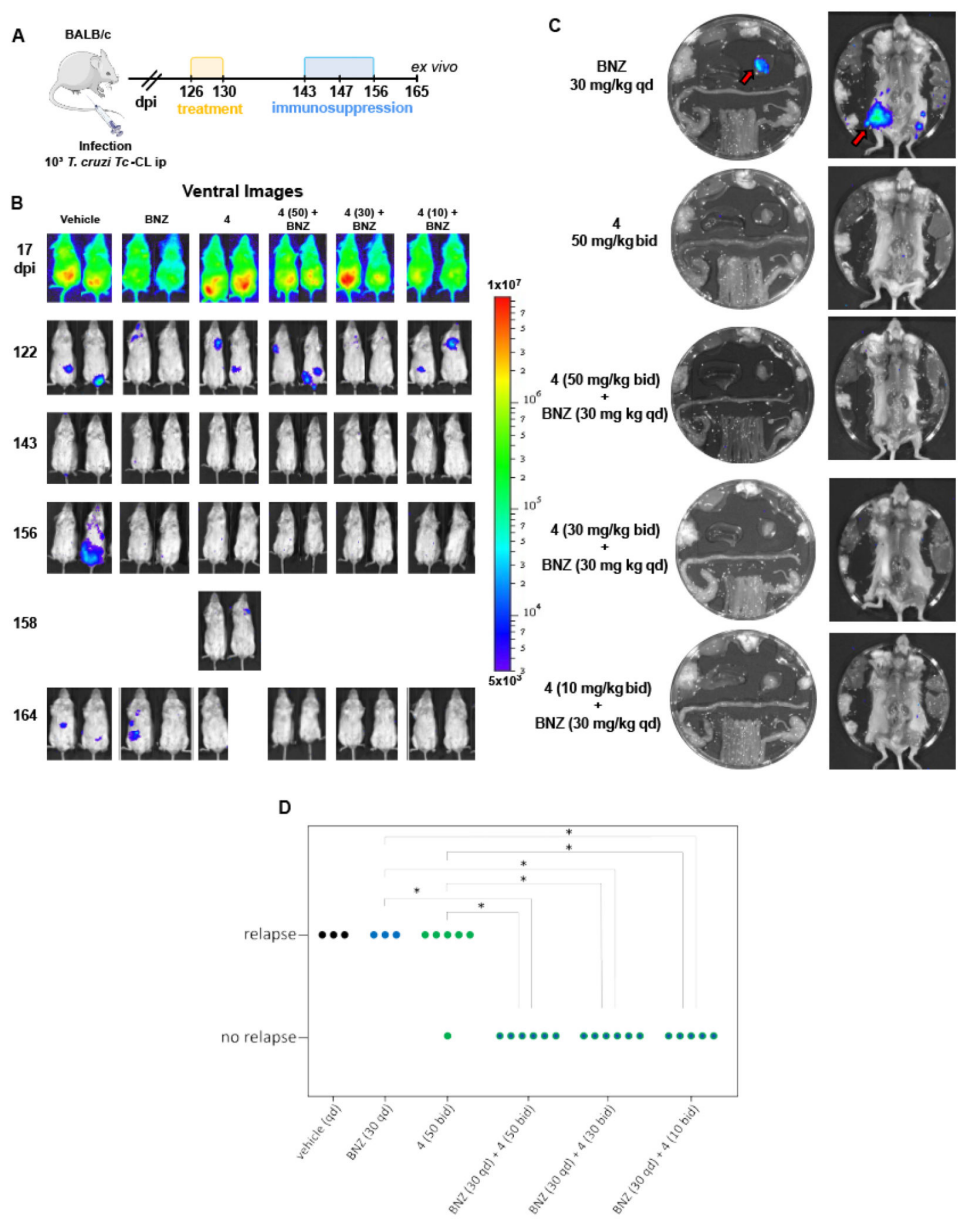
*In vivo* assessment of the combination of compound 4 with a low dose of BNZ in a murine model of chronic *T. cruzi* infection. (A) Schematic representation of experiment. Parts of the figure were drawn by using pictures from Servier Medical Art. Servier Medical Art by Servier is licensed under a Creative Commons Attribution 3.0 Unported License (https://creativecommons.org/licenses/by/3.0/).” Bioanalysis data for this experiment is shown in Table S5. (B) Representative ventral images of BALB/c mice at the chronic stage of infection (126 days) treated orally for 5 days with vehicle (once daily (qd), n = 3), BNZ (30 mg/kg, qd, n = 3), compound 4 (50 mg/kg, twice daily (bid), n = 6), BNZ (30 mg/kg, qd) in association with compound 4 (30 mg/kg, bid, n = 6) and BNZ (30 mg/kg, qd) in association with compound 4 (10 mg/kg, bid, n = 5). Bioluminescent-negative mice were immunosuppressed on days 143, 147 and 156 using cyclophosphamide (200 mg/kg i.p.). For welfare reasons, we did not continue immunosuppression if there were definitive indications of a relapse. (C) At day 165 post-infection, organs of non-relapsed animals were removed and assessed for bioluminescence. Representative *ex vivo* images of organs (upper) and carcass (lower) are shown. For *ex vivo* imaging, organs, tissues, and carcass were displayed as in Fig. 3. Red arrows indicate bioluminescence foci. All mice treated with compound 4 in association with BNZ, were assessed as cured (d and e). Heat-maps are on log10 scales and indicate intensity of bioluminescence from low (blue) to high (red). (D) Outcomes plot: mice were designated as not relapsed only when bioluminescent negative in both *in vivo* and *ex vivo* imaging. Outcome for each animal plotted for each treatment. Fisher’s exact test was used to calculate statistical significance (α = 0.05). 95% confidence intervals for no-relapse were calculated with the Adjusted Wald method (85) and are as follows: BNZ (30 qd) [0.06 − 0.8], 4 (50 bid) [0.01 − 0.58], BNZ + 4 (50 bid) [0.64 − 1], BNZ + 4 (30 bid) [0.64 − 1], BNZ + 4 (10 bid) [0.6 − 1]. Treatment colours: vehicle = black, BNZ = blue, cpd 4 monotherapy = green, BNZ + cpd 4 combinations = blue/green. Treatment regimens on y axis are for 5 days and expressed as mg/kg).

The co-administration of compound 4 (at 50, 30 or 10 mg/kg bid) with BNZ at the known suboptimal dose of 30 mg/kg (qd) prevented relapse in all treated animals, whereas all monotherapy conditions showed frequent relapses (Fig. 6). These results were statistically significant and indicate that co-administration of a pyrrrolopyrimidine with BNZ has potential for a short-course treatment strategy for Chagas disease. Overall, our strategy has enabled the identification of compound 4 as a selective *T. cruzi* complex III inhibitor which provides full efficacy against experimental chronic Chagas disease when dosed orally for 5 days in combination with BNZ.

## Discussion

Our aim is to develop new treatments for Chagas disease with the potential to eradicate all parasites from patients, thus providing the best chance of preventing the debilitating pathology (48). Here we demonstrate that combination treatment of cytochrome *b* inhibitors and a low dose of BNZ, the current first-line treatment (49), results in full efficacy in our mouse model of chronic Chagas disease in a short five-day treatment regimen. These findings offer the prospect of a short-course clinical treatment that aligns with the ideal Target Product Profile for Chagas disease (14) as it may overcome both efficacy and safety challenges with the current treatments. As such this combination approach could be truly transformative for Chagas disease patients. There is abundant precedent for the use of combination therapy across viral (50), bacterial (51) and parasitic (52) infections as a valuable way of increasing efficacy, preventing resistance or shortening treatment regimens. In addition, combination of BNZ with cytochrome *b* inhibitors may allow reduction of the BNZ dose below toxic concentrations. Assessment of combined treatment of antiparasitic drugs in human Chagas disease has been limited and clinical trials combining BNZ with CYP51 inhibitors have shown no benefit over BNZ monotherapy, highlighting the need to explore better combination partners (53–61).

Our results raise several interesting questions. First, how will this translate to the clinic? In our studies we have chosen 30 mg/kg qd for BNZ in combination studies as this is substantially lower than the minimum efficacious dose in a five-day chronic study (100 mg/kg) (62). To identify the appropriate dose in the clinic, multiple doses will likely need to be tested in clinical trials, based on human dose predictions. Despite the identification of more selective *T. cruzi* cytochrome *b* inhibitors with lower propensity for effects on mammalian mitochondria *in vitro*, mitochondrial toxicity *in vivo* remains an important concern. Potential dose reduction in combination treatments may minimize the mitochondrial toxicity risk. A second key question is why this combination offers such rapid clearance of parasites, unlike combinations of BNZ with CYP51 inhibitors. Our *in vitro* results showing increased rate-of-kill for the combination over the respective monotherapies offer a first insight, but more work is required to understand the underlying molecular basis. Furthermore, the failure of *T. cruzi* cytochrome *b* Qi site inhibitors, which are frequently identified (63, 64), to prevent relapse as monotherapy, does not preclude that longer treatment regimens may achieve full elimination of all parasites. However, there are important benefits to short duration treatment regimens. Interestingly, the pyrrolopyrimidine series also shows promise as a new treatment for visceral leishmaniasis, which is caused by the related parasite *Leishmania donovani*, as shown elsewhere in this issue (65).

A key purpose of *in vitro* assays in drug discovery is to predict outcomes *in vivo* (and eventually the clinic). This has been a challenging area in Chagas disease drug discovery, where many assays show a favorable profile for compounds that eventually fail *in vivo*, despite excellent pharmacokinetics. One explanation is the presence of a small subpopulation of parasites that is less susceptible to drugs, so called persisters, which cannot be detected by most *in vitro* assays. Here we present data to support the predictive power of washout outgrowth assays for Chagas disease drug discovery. Monotherapy treatment with the pyrrolopyrimidine cytochrome *b* inhibitors could not achieve full cure in our *in vitro* washout assay (as seen for the unrelated *T. cruzi* cytochrome *b* inhibitor GNF7686 (66)), predicting the *in vivo* relapses, while combination treatment with BNZ did prevent relapse, both *in vitro* and *in vivo*. Designing washout experiments, and in particular combination experiments, is challenging with multiple factors, such as parasite numbers, doses for the respective compounds and washout duration, to consider. In terms of dose selection for monotherapy arms we opted to use high concentrations of the cytochrome *b* inhibitors (50-fold EC_50_) to assess the effect of maximum inhibition of the target. For the combination experiments our rationale was to use ineffective concentrations for both partners while maintaining total drug pressure (50-fold EC_50_) equal to the cytochrome *b* monotherapy arms. The need for a long post-washout culture phase is exemplified by our detection of relapses as late as 32 days post washout.

In summary, we have identified a highly selective *T. cruzi* cytochrome *b* inhibitor, that in combination with a reduced dose of BNZ, achieves full efficacy in a clinically predictive mouse model of chronic Chagas disease. These results offer an opportunity for a transformative short course treatment for Chagas disease patients and warrant progressing this combination further towards the clinic.

## Materials and Methods

### Study design

The objective of this study was to develop new potential treatments for Chagas disease. Through unbiased screening of a large, diverse chemical library we identified a compound with promising activity against *Trypanosoma cruzi* parasites. Hit compound and derivatives were profiled in a panel of assays in at least three independent replicates. Mode of action studies were carried out on two compounds from the pyrrolopyrimidine series. Resistance generation studies were carried out in three independent replicates. Using randomly selected mice we tested the efficacy of the compounds and combinations in a well-established model for chronic Chagas disease. Fisher’s exact test was used to assess significance, with *P* < 0.05 considered significant. No statistical methods were used to predetermine the sample size. The investigators were not blinded to the allocation during experiments and outcome assessment. Data output was in the form of both optical images and bioluminescence flux (photons/sec/cm2), which reduces the scope for observer bias.

### *In vitro* parasite assays

Parasite culture and potency determinations against intracellular *T. cruzi* were carried out as described in (19, 20) with the only modification that treatment duration was 96 hours instead of 72 hours. Trypomastigote assays using strain Silvio X10/7 subclone A1 were carried out as described in (19). Parasite culture and potency determinations for panel of strains were performed as in (19). Strains used were M6241 clone 6 (DTU III), ERA clone 2 (DTU IV), PAH179 clone 5 (DTU V) and CL Brener-Luciferase (PpyRE9h-expressing *T. cruzi*, same strain that is used in animal model studies (67)) (DTU VI). Compound exposure time was 5 days for M6241, ERA, PAH179 and CL Brener-Luciferase. For Y strain compound exposure time was 3 days. Rate-of-kill assays were carried out as described in (39).

Washout outgrowth assay: Vero cells were infected overnight in T600 flasks at a multiplicity of infection (MOI) 5 with *T. cruzi* Silvio X10/7 A1 trypomastigotes, followed by washing of the Vero monolayer to remove free trypomastigotes. 24 hours later the monolayer was trypsinised, 5.3 x 10^6^ Vero cells were plated in T75 tissue culture flasks (in 20 ml MEM-Glutamax (ThermoFisher), 1% Fetal Calf Serum (FCS)), and treatment was started. For ten-day treatments, medium with compounds was replaced after the initial 5 days. For 16 day treatments the compounds were replaced every four days. For compound washout, all medium was aspirated, and flasks were washed three times with 10 ml serum-free MEM medium followed by a final aspiration and addition of 20 ml MEM-Glutamax (ThermoFisher) supplemented with 1% FCS. The cultures were then maintained until trypomastigotes were detected by light microscopy. Medium was replaced twice weekly and prior to each medium replacement microscopic evaluation was carried out (five fields per flask) for up to 60 days post washout. Robustness of the data was confirmed by profiling multiple compounds from the pyrrolopyrimidine series in a single experiment (compounds 2,3 and 4 in Fig. 2 and Fig. S17) as well as comparing results from independent experiments (compound 2 in Fig. 2, Fig. S5 and Fig. S7).

### *T. cruzi* CYP51 assay

As described in (68).

#### General cytotoxicity assay in HepG2 cells

This assay was previously described (36). Briefly, actively growing HepG2 cells were removed from a T-175 TC flask using 5 ml Eagle’s MEM (containing 10 % FBS, 1 % NEAA, 1 % penicillin/ streptomycin. Carbon source is D-glucose at 1 g/l) and dispersed in the medium by repeated pipetting. Seeding density was checked to ensure that new monolayers were not more than 50 % confluent at the time of harvesting. Cell suspension was added to 500 ml of the same medium at a final density of 1.2 x 10^5^ cells/ml. This cell suspension was dispensed (25 μl, 3000 cells per well) into 384-well clear-bottom plates using a Multidrop Combi dispenser. Prior to addition of the cell suspension, the screening compounds (250 nl) were predispensed into the plates with an EchoH liquid handler. Plates were incubated for 48 h at 37°C, 5% CO_2_. After incubation, plates equilibrated at room temperature for 30 min before proceeding to develop the luminescent signal. The signal developer, CellTiter-Glo Reagent, was allowed to equilibrate at room temperature for 30 min and added to the plates (25 ml per well) using a Multidrop Combi dispenser. Plates were left for 10 min at room temperature for stabilization and then read using a ViewLux.

### Mitotoxicity assays

#### Measurement of cell bioenergetics in HepG2 cells.

To measure the Oxygen Consumption Rate (OCR) in cells following compound treatment, the Seahorse XFe96 Extracellular Flux Analyser (Agilent) was used (69). HepG2 cells were maintained in DMEM medium (Invitrogen #11966-025) supplemented with 10 mM galactose, 1 mM sodium pyruvate, 5 mM HEPES, 2 mM GlutaMAX, 10% fetal bovine serum and 50 μg/ml gentamycin. Cells were maintained in a 37 °C, 5% CO_2_ humidified atmosphere. The measurement of OCR was performed as previously described (70, 71) with minor modifications. Briefly, HepG2 cells were seeded in an XF96-well plate at 14000 cells per 100 μl cell culture medium per well and incubated in a 37 °C, 5% CO_2_ humidified atmosphere for 24 hours. Following incubation, the culture medium was replaced with 175 μl pre-warmed, serum-free XF base assay medium (Agilent), supplemented with 2 mM *L*-glutamine, 1 mM sodium pyruvate and 10 mM galactose (pH 7.4) and incubated at 37 °C for one hour. Test compounds (compound 3 and 4) were maintained in DMSO and a four-point dose response (final concentrations 10, 20, 40 and 80 μM), plus 0.4% DMSO control, were diluted to 8× concentration in XF assay medium and 25 μL was loaded into Port A of the XF Assay Cartridge. The mito-stress test components oligomycin (1 μM final), carbonyl cyanide-4-(trifluoromethoxy)-phenylhydrazon [FCCP] (0.8 μM final) and antimycin A/rotenone (1 μM final) were loaded into ports B, C and D at 9×, 10× and 11× concentrations respectively (all 25 μl volume). Following calibration of the XF Assay Cartridge, the cell plate was inserted into the analyzer and 2 basal measurements were made using a 3-minute mix, 3-minute measure protocol. Compounds 3 or 4 were then injected into each well from port A. The mito-stress test components were injected into each well from ports B-D with three, 3-minute mix, 3-minute measure cycles performed between each injection. Each experimental run consisted of 2 baseline measurements before the addition of the test compounds, followed by 10 basal OCR measurements after the addition of the test compounds. Consecutively, 3 more measurements were made after the addition of each of the mito-stress test components. The OCR data used for the analysis were collected from measurement 12 (the 10th basal OCR measurement following test compound addition). The lowest effective concentration (LEC) was estimated for each compound based on the test concentration causing a reduction in oxygen consumption rate ≥15% compared to DMSO control in at least 2 out of three replicates.

#### Human complex III inhibition assay

Preparation of mitochondria from a human cell line (THP-1) was performed following published procedure (72). The protocol for the assay has been described in (73).

#### Calcium Loading Capacity (CLC) assessment in rat liver mitochondria

The CLC of freshly isolated liver mitochondria was determined as previously described (70). Briefly, mitochondria were suspended at 0.2 mg/ml in incubation buffer B, supplemented with 100 μM arsenazo III, 2.5 μM rotenone and 1 μg/ml oligomycin in each well of a 96 well microplate (control and treated mitochondria were assayed simultaneously) and absorbance measured every 2 seconds at 650 nm in a FlexStation3 microplate reader (Molecular Devices, Sunnyvale, CA, USA). After approximately 1 minute of reading, Ca^2+^ was added (0.5-1 μmol/ mg mitochondrial protein) followed by the addition of 10 mM succinate as the mitochondrial substrate. The CLC was calculated as the difference in optical density (ΔOD) between the point at which mitochondria were energized and the point where mitochondrial permeability transition was triggered (*i.e*., the lowest OD reading before calcium release via MPT).

The concentration-response relationship was modelled using a robust fit of a cubic polynomial regression (74, 75). The fitted curves were then used to calculate the Minimum Effective Concentration (MEC). The MEC was defined as the lowest concentration where the fitted curve exceeded 3 standard deviations (SD) around the DMSO mean response. SD was estimated from the deviations of DMSO and compound treated wells around the fitted means, using a robust estimator based on Gini’s mean difference (76).

### *In vivo* efficacy with bioluminescence assay and bioanalysis

Experiments followed a similar protocol to that described in (21). All animals were female (BALB/cAnNCrlC mice, 6-8 weeks old, strain code: 028, supplier: Charles River (UK)). We have previously shown that there are no differences in the bioluminescence profiles between infected males and females (77). Animals were maintained under specific pathogen-free conditions in individually ventilated cages. They experienced a 12-hour light/dark cycle, with access to food and water ad libitum. Mice were randomly selected and distributed into cages on arrival day by a Named Animal Care and Welfare Officer (NACWO), at LSHTM biological service facility (BSF). For efficacy experiments animals were randomly selected from the cages and ear/tail tagged on the infection day, starting from number 1. The same mouse ID was kept until the experimental endpoint. Mice were infected by i.p injection with 1x10^3^ trypomastigotes derived from SCID mouse blood (67). To confirm infection, mice were imaged around the peak of parasite load. Mice that did not show the expected levels of bioluminescence were excluded (humanely killed) from the experiment. Treatment initiated in the chronic stage of infection ranging between 90 and 127 days post-infection. Drugs were administered by oral gavage (~200 μl), and vehicle only (0.5% w/v hydroxypropylmethylcellulose with 0.4% v/v Tween 80 and 0.5% v/v benzyl alcohol) was administered to control mice. Total number of animals for each study arm is shown in the relevant figures. To detect residual infection, mice were immunosuppressed using cyclophosphamide (at 200 mg/kg) by ip injection every 4 days for a maximum of three doses. For *in vivo* imaging, mice were injected ip with 150 mg/kg D-luciferin in Dulbecco’s Ca^2+^/Mg^2+^-free PBS, then anaesthetized using 2.5% (v/v) isofluorane in oxygen. Images were obtained using an IVIS Lumina II system (Caliper Life Science) 10−20 min after D-luciferin administration. The detection threshold was established from uninfected mice. Organs/tissue samples were assessed by *ex vivo* imaging as described previously (21). The limit of detection in this system is less than 20 parasites (29).

Following the first daily dose on day 1 and last day of treatment, serial blood samples were taken by the tail vein from all treated mice pre-dose, then 30 minutes, 1, 2, 4, and 8 hours post-dose, mixed with two volumes of distilled water and analyzed for test compound. Quantification of compounds was performed by means of LC/MSMS and exposure parameters namely C_max_ and AUC_last_ were estimated using Phoenix 64 (Pharsight Certara).

Animal work was performed under UK Home Office project licenses (PPL 0/8207 and PPL P9AEE04E4) and approved by the London School of Hygiene and Tropical Medicine Animal Welfare and Ethical Review Board. Procedures were performed in accordance with the UK Animals (Scientific Procedures) Act 1986 and the GSK Policy on the Care, Welfare and Treatment of Animals

### *In vitro* potency combination experiments

For combination interaction analysis a full dose-response matrix experiment was set up with 11 concentrations of pyrrolopyrimidine series compound (1 μM top concentration, 1:3 dilution) against 8 concentrations of BNZ (50 μM top concentration, 1:3 dilution) in an intracellular *T. cruzi* assay (78). Two independent experiments were performed and the number of amastigotes per host cell at 72 hours post infection was used as parameter to determine interactions between the compound pairs using Genedata Screener software. For each pair an isobologram was calculated and goodness of fit was determined (reduced chi-square statistic). For interaction analysis the fractional combination index was calculated using the Chou-Talalay method (79).

### Cell lines and culture conditions − mode of action studies

*T. cruzi* epimastigotes from the Silvio strain (MHOM/BR/78/Silvio; clone X10/7A) were grown at 28°C in RTH/FBS medium (80).

### Drug sensitivity assay − mode of action studies

*T. cruzi* epimastigote assays carried out as described previously (43).

### Generation of drug-resistant parasites

Compound-resistant cell lines were generated by subculturing clones of wild-type *T. cruzi* epimastigotes in the continuous presence of compound 3 or compound 5. Starting at sublethal concentrations, drug concentrations in 3 independent cultures were increased in a stepwise manner, usually by 2-fold. When parasites were able to survive and grow in compound concentrations equivalent to >30-fold their established EC_50_ value, they were cloned by limiting dilution in the presence of compound. Three independent clones were selected from each compound for further study.

### Whole genome sequencing

Genomic DNA was purified from *T. cruzi* compound-sensitive (wild-type clone) and cloned resistant parasites (~1 x 10^8^) using a standard phenol-chloroform/alkaline lysis protocol. Whole genome sequencing was performed using PCR-free library construction and the DNBseq next generation sequencing platform (Beijing Genomics Institute, Hong Kong). Sequencing reads (150 bp paired end) were aligned to the *T. cruzi* Dm28c 2018 genome sequence (v46; tritrypdb.org) alongside the maxi-circle sequence (FJ203996.1, NCBI). Reads were aligned using Bowtie2 using the settings “very-sensitive” and Samtools software (81, 82). Single nucleotide polymorphisms (SNPs) and indels were called using BCFtools (mpileup) where overall quality score (QUAL) was >100 when compared with the wild-type clone (83). Chromosome and gene copy number variation (CNV) analysis was performed using Artemis (84). Median read counts of the wild-type and resistant clones were used to normalize copy number. Data sets have been deposited with the European Nucleotide Archive (accession number: **PRJEB47248**). Cytochrome *b* genes from resistant parasites were also Sanger sequenced in-house using primers: 5′-GAGAGAGAGTTTCGAGAGGGA-3′ (forward) and 5′TCTAAATTCGCCCAAATTCCTCTTA-3′ (reverse).

### *T. cruzi* complex III assay

Measurement of complex III activity and inhibition were performed as described previously (43).

#### Protein and ligand preparation for molecular modelling

Our molecular modelling studies were based on a previously generated homology model of *T. cruzi* cytochrome *b* (43). The protein structure with a conserved water molecule interacting with F33 was prepared using the Protein Preparation module in the Schrödinger suite (Schrödinger Release 2021-2). Protonation states were assigned by PROPKA at pH 7.0, and the hydrogen bonding network was consequently optimized. A restrained energy minimization step was then executed using the OPLS4 force field with default settings. Models of cytochrome *b* bearing either L197I or F222L mutations were prepared in Maestro by mutating one residue at a time. Subsequently, the mutated versions of cytochrome *b* were processed in with the Protein Preparation tool in an identical manner to the wild-type enzyme.

Ligand structures (compounds 1-4) were prepared with Schrödinger’s LigPrep (Schrödinger Release 2021-2). All possible tautomeric forms and stereoisomers were generated at pH 7.0 ± 0.4 using Epik.

### Docking studies

Molecular docking studies were carried out using Glide Standard Precision (SP) mode (Schrödinger Release 2021-2). First, the docking grid boxes were generated using the Receptor Grid Generation tool; bound ubiquinone (UQ2) was selected as the center of the grid, and a cube of 10 Å was defined as the inner box. During docking, a total of 20 poses per ligand were subjected to post-docking minimization and a maximum of ten docking poses for each ligand were output. This setup was able to successfully reproduce the experimentally-determined binding mode of antimycin A.

### Molecular dynamic simulation

Molecular Dynamic (MD) simulation of the binding mode generated by molecular docking was carried out to analyze the stability of the pose and the interactions at the binding site. The docking pose of compound 3 in complex with *T. cruzi* cytochrome *b* was used as the initial coordinate for the generation of the MD system. Desmond software (Schrödinger Release 2021-2) was utilized to set up the system and run the MD simulation. As cytochrome *b* is embedded in the inner mitochondrial membrane, MD was performed in membrane; thus, 1-palmitoyl-2-oleoyl-sn-glycero-3-phosphocholine (POPC) membranes were added to the model system by placing the two layers automatically. The system was then solvated using the SPC (simple point-charge) water model in a Periodic Boundary Conditions orthorhombic box and neutralized with Na^+^ ions at a salt concentration of 0.15 M. The option “exclude ions and salt placement” was turned on to allow a 10 Å buffer zone from the ligand. The default Desmond protocol for energy minimization and model relaxation was applied before performing the production simulation. The OPLS4 force field and NPAT (constant particle number (N), pressure (P), lateral surface area (A), and temperature (T)) ensemble were used. The temperature was kept constant at 300 K, while the pressure at 1.01325 bar. Lastly, a 100 ns MD simulation with a trajectory interval of 5 ps was carried out with the same conditions. Simulation Interactions Diagram (SID) was used for the analysis of the MD simulation. The RMSF and RMSD values were then plotted using R package.

## Materials and Methods

### Chromatographic LogD assay

The chromatographic hydrophobicity index (CHI) values were measured using reversed phase HPLC column (50 mm × 2 mm, 3 μm Gemini NX C18, Phenomenex, U.K.) with fast acetonitrile gradient at starting mobile phase at pH 7.4. CHI values were derived directly from the gradient retention times by using a calibration line obtained for standard compounds. The CHI value approximates to the volume % organic concentration when the compound elutes. CHI was linearly transformed into ChromlogD by least-squares fitting of experimental CHI values to calculated log P (ClogP) values for over 20K research compounds using the following formula: ChromlogD = 0.0857CHI − 2.00. The average error of the assay is ±3 CHI units or ± 0.25 ChromlogD.

### Kinetic aqueous solubility

The aqueous solubility of test compounds was measured using an in-house method utilizing quantification via chemiluminescent nitrogen detection (CLND): a 5 μL of 10 mM DMSO stock solution was diluted to 100 μL with pH 7.4 phosphate buffered saline and equilibrated for 1 h at rt and filtered through Millipore Multiscreen HTS-PCF filter plates (MSSL BPC). The eluent is quantified by suitably calibrated flow injection CLND. This assay has a dynamic range between the lower detection limit of 1 and 500 μM, governed by the protocol’s 1:20 dilution into pH 7.4 phosphate buffer solution from nominal 10 mM DMSO stock.

### FaSSIF solubility assay

Solubility of test compounds in fasted simulated intestinal fluid (FaSSIF) was determined at pH 6.5 after 4 h equilibration at rt. An amount of1 mL of FaSSIF buffer (3 mM sodium taurocholate, 0.75 mM lecithin in sodium phosphate buffer at pH 6.5) was added to manually weighed 1 mg of solid compound in a 2 mL HPLC autosampler vial. The resulting suspension was shaken at 900 rpm for 4 h at rt and then transferred to a Multiscreen HTS, 96-well solubility filter plate. The residual solid was removed by filtration. The supernatant solution was quantified by HPLC−UV using single-point calibration of a known concentration of the compound in DMSO. The dynamic range of the assay was 1−1000 μg/mL.

### Intrinsic clearance (Cl*int*) in microsomes

Intrinsic clearance (CL*int*) values were determined in male CD-1 mouse and human liver microsomes (0.5 μM). Immediately at time zero and at several serial times points up to 30 min, an aliquot of the incubation mixture was removed and mixed with acetonitrile to stop the reaction. Internal standard was added to all samples, the samples centrifuged to sediment precipitated protein and the plates then sealed prior to HPLC-MS/MS analysis. Metabolic stability expressed as a percentage of the parent molecule was calculated using the peak area ratio (compound peak area/internal standard peak area) of different compounds remaining after each incubation time compared to time zero of the incubation. The half-life (t1/2) was calculated using the following equation:

t1/2=−ln(2)/k,

where k is the turn-over rate constant of the ln (percent remaining) vs time regression.

CL*int* was calculated from the half-life (t1/2) using the following equations:

CL*int* (ml/min/g liver) = (0.693/(t1/2) x (ml of incubation/mg microsomal protein) x (mg microsomal protein/g liver).

### Plasma protein binding

*In vitro* plasma protein binding was measured in mouse and human plasma using equilibrium dialysis at a nominal concentration of 2 μM. The RED inserts were placed in the 48 wells of the Teflon Plate (Pierce). Samples were prepared by mixing the test compound with plasma at the appropriate concentrations to yield a final drug concentration of 2 μM. Triplicate aliquots of plasma containing compounds at a concentration of 2 μM were pipetted to plasma side (red) of the insert, and PBS (phosphate buffered saline) pH 7.4 was placed into the receiver side (white) of the insert. The plate was covered with the sealing tape and incubated in a 37 °C orbital shaker water bath at approximately 150 rpm for 4 h. Following incubation, samples were prepared in a mixed matrix configuration. Aliquots of samples were pipetted into 96-well plates, and precipitation buffer was added to protein precipitate the samples. Samples were vortexed to mix, then centrifuged for 15 min at 3700 rpm and 4 °C. The supernatant was assayed directly by LC−MS/MS. The following equation was used to calculate the percentage bound drug fraction using this equilibrium dialysis method

% unbound = (buffer chamber]/[plasma chamber) x100

% PPB = 100 - % unbound

### Blood to plasma ratio

The extent of association of compounds with blood cells was measured in vitro using mouse, and human blood. Untreated whole blood was pre-warmed on a rotary shaker at 37°C. Samples were prepared by mixing test compound with whole blood to yield a final drug concentration of 1 μM. Once mixing was complete, at T(0) and T(30 min), aliquots of incubated blood containing different compounds were mixed with milliQ water. To precipitate the samples for analysis, precipitation buffer containing Internal Standard (is), were added to the individual samples. Samples were centrifuged for 10 min at 15,000 × g. The supernatant was assayed directly by LC-MS/MS. The following equation was used to calculate the blood to plasma partitioning ratio: Blood:plasma ratio = [blood]/[plasma] Compound: IS peak area ratios are used as representative of the relative compound concentrations in the blood and plasma samples.

### Synthesis

All starting materials were purchased from commercial sources and used as received. Solvents were dried using a commercial solvent purification system and stored under nitrogen. All final compounds were characterized by ^1^H NMR spectroscopy and LCMS.^1^H NMR spectra were recorded on a Bruker Advance 400 MHz spectrometer at 25°C. The internal standard used was the residual protonated solvent at 7.30 ppm for CDCl3, 3.31 and 4.89 for CD3OD, 2.50 ppm for DMSO-*d*6. Chemical shifts for protons are reported in parts per million (ppm). Abbreviations used to identify the splitting or coupling patterns were described as s (singlet), d (doublet), dd (doublet of doublets), t (triplets), td (triplet of doublets), q (quartet), m (multiplet) and br (broad), sep (septet). Coupling constants (*J*) are quoted to the nearest 0.1 Hz. Software for acquisition: Topspin 1.3. Software for visualization: ACD SpecManager v12.5. LC-MS analyses were performed with either an Agilent 1100 series HPLC connected to a Bruker Daltonics MicrOTOF, or an Agilent Technologies 1200 series HPLC connected to an Agilent Technologies 6130 quadrupole LC/MS, where both instruments were connected to an Agilent diode array detector. Mobile phase was water/acetonitrile + 0.1% HCOOH, or water/acetonitrile + 0.1% NH3; linear gradient 80:20 to 5:95 over 3.5 min, and then held for 1.5 min; flow rate 0.5 ml/ min. Mass spectra were obtained using electrospray ionization (ESI) techniques.

All final compounds submitted to biological profiling showed a purity of ≥ 95%, unless otherwise noted. Purity was determined by HPLC (Acquity UPLC BEH C18 1.7 μm, 2.1 mm × 50 mm, ammonium acetate 25 mM + 10% acetonitrile at pH 6.6/acetonitrile) at 35 °C. Samples prepared at 0.1 mg/ml concentration in methanol.

### Synthesis of compounds 1,2,3,4

#### Step 1, Intermediate B

2-Amino-6-methyl-3,7-dihydro-4*H*-pyrrolo[2,3-*d*]pyrimidin-4-one. Sodium acetate (45.5 g, 555 mmol) was added to a suspension of 2,6-diaminopyrimidin-4(5*H*)-one (70g, 555 mmol) in water (1402 ml) and the mixture was heated to reflux. 1-chloropropan-2-one (55.0 ml, 666 mmol) was added in three portions (each portion every 30 minutes) and the mixture was stirred at reflux for 3.5 hours. The reaction mixture was cooled to room temperature and then kept at 4 °C for 16 hours. The resulting precipitate was filtered, washed with cold water and dried *in vacuo* to yield 62 g (68.0 % yield) of title compound as a beige solid. ^1^H NMR (400 MHz, DMSO-*d*6) δ ppm 10.88 (br s, 1 H), 10.30 (br s, 1 H), 6.16 (br s, 2 H), 5.78 - 5.95 (m, 1 H), 2.15 (d, *J* = 1.0 Hz, 3 H). LC-MS (ESI): m/z 165; [M+H]+.

#### Step 2, Intermediate C

4-Chloro-6-methyl-7*H*-pyrrolo[2,3-*d*]pyrimidin-2-amine. *N,N*-dimethylaniline (0.96 ml, 7.6 mmol) was added to a stirred solution of 2-amino-6-methyl-3,7-dihydro-4*H*-pyrrolo[2,3-*d*]pyrimidin-4-one (31 g, 191 mmol in phosphorus oxychloride (319 ml, 3435 mmol) in a 5l reactor and the mixture was stirred at 110 °C for 3 hours. After cooling to room temperature, the mixture was slowly poured on crushed ice and was slowly neutralized with aqueous ammonia at 0°C. Precipitate formed was filtered and washed with cold water dried *in vacuo* (with addition of toluene) to yield title compound as a pale grey solid (20.2 g, 58 % yield). ^1^H NMR (400 MHz, DMSO-*d*6) δ ppm 11.33 (br s, 1 H), 6.36 (s, 2 H), 5.93 (m 1 H), 2.26 (d, *J* = 1.0 Hz, 3 H). LC-MS (ESI): *m/z* 183, 185; [M+H]^+^, 1x Cl isotopic pattern observed.

#### Step 3, Intermediate D

*N*-(4-Chloro-6-methyl-7*H*-pyrrolo[2,3-*d*]pyrimidin-2-yl)pivalamide. Trimethylacetyl chloride (pivaloyl chloride) (3.37 ml, 27.4 mmol) was added dropwise to a stirred solution of 4-chloro-6-methyl-7*H*-pyrrolo[2,3-*d*]pyrimidin-2-amine (4 g, 22 mmol) and pyridine (17.5 ml) and the resulting mixture was stirred for 3 hours at room temperature. The reaction mixture was diluted with water and extracted several times with a 20% mixture of methanol in dichloromethane. The combined organic phases were washed with brine, dried over sodium sulfate (Na_2_SO_4_), filtered and solvents evaporated *in vacuo*. The residue was triturated with diethylether to provide title compound (3.76g, 64% yield) as a light brown solid. ^1^H NMR (400 MHz, CDCl3) δ ppm 10.37 (br s, 1 H); 8.23 (s, 1 H), 6.26-6.28 (m, 1 H), 2.45 (d, *J* = 1.0 Hz, 3 H), 1.42 (s, 9 H). LC-MS (ESI): *m/z* 267, 269; [M+H]^+^, 1xCl isotopic pattern observed.

#### Step 4, Intermediate E

*N*-(4,6-Dimethyl-7*H*-pyrrolo[2,3-*d*]pyrimidin-2-yl)pivalamide. The reaction was performed in 7 batches. A solution of trimethylaluminium toluene (2 M, 244 ml, 487 mmol) was added to a mixture of *N*-(4-chloro-6-methyl-7*H*-pyrrolo[2,3-*d*]pyrimidin-2-yl)pivalamide (65 g, 244 mmol) and tetrakis(triphenylphosphine)palladium (56.3 g, 49 mmol) in tetrahydrofuran (THF) (6716 ml) and the mixture was stirred at 75 °C for 16 hours. Saturated aqueous ammonium chloride (NH4Cl) was added slowly to quench the reaction. The reaction batches were combined and the mixture was filtered through a celite pad and washed with ethyl acetate (EtOAc). Water was added to the washings and the layers were separated. The organic phase was washed with water and concentrated. The residue was dissolved in hydrochloric acid (HCl 2N) and washed with EtOAc four times. The aqueous phase was basified with sodium carbonate (Na2CO3) until pH 12 and extracted with EtOAc three times. The combined organic phases were dried over Na_2_SO_4_, filtered and the solvents evaporated *in vacuo*. The residue was dissolved in diethyl ether. The resulting precipitate was filtered and washed with diethyl ether to yield title compound as a yellow solid (45 g, 75 % yield). ^1^H NMR (400 MHz, CDCl3) δ 9.89 ppm (br s, 1 H), 8.10 (s, 1 H), 6.12-6.15 (m, 1 H), 2.60 (s, 3 H), 2.37 (d, *J* = 1.0 Hz, 3 H)1.35 (s, 9 H). LC-MS (ESI): *m/z* 247; [M+H]^+^.

#### Step 5, Intermediate F

*N*-(5-Iodo-4,6-dimethyl-7*H*-pyrrolo[2,3-*d*]pyrimidin-2-yl)pivalamide. A mixture of *N*-(4,6-dimethyl-7*H*-pyrrolo[2,3-*d*]pyrimidin-2-yl)pivalamide (45 g, 183 mmol) was dissolved in tetrahydrofuran (THF) (406 ml) and a mixture of *N*-iodosuccinimide (82 g, 365 mmol) dissolved in THF (203 ml) was slowly added. The mixture was stirred for 2 hours at 25 °C The mixture was treated with water and ethyl acetate. The organic layer was separated and washed with a saturated aqueous sodium bisulfite solution followed by brine. The organic layer was evaporated *in vacuo* to yield title compound (35 g, 52 % yield) as a brown solid. ^1^H NMR (400 MHz, CDCl3) δ 9.48 ppm (br s, 1 H), 8.10 (br s, 1 H), 2.88 (s, 3 H), 2.40 (s, 3 H), 1.35 (s, 9H). LC-MS (ESI): *m/z* 373; [M+H]^+^.

#### Step 6, Intermediate G

*N*-(5-Iodo-4,6-dimethyl-7-tosyl-7*H*-pyrrolo[2,3-*d*]pyrimidin-2-yl) pivalamide. *N*-(5-iodo-4,6-dimethyl-7*H*-pyrrolo[2,3-*d*]pyrimidin-2-yl)pivalamide (3g, 8 mmol) was dissolved in tetrahydrofuran (THF) (17 ml) and sodium hydride, 60% dispersion in mineral oil (967 mg, 24.2 mmol) was added at 0 °C. The mixture was stirred for 15 minutes and then, 4-methylbenzene-1-sulfonyl chloride (4.6 g, 24.2 mmol) in solution in THF (10 ml) was added and the mixture was stirred for 16 hours at room temperature. The mixture was diluted with EtOAc and was washed with aqueous sodium carbonate and brine. The organic phase was dried over Na_2_SO_4_, filtered and concentrated *in vacuo*. The residue was purified by flash column chromatography on silica gel eluting with a gradient 0-40% EtOAc/cyclohexane. The fractions containing product were collected and the solvents evaporated *in vacuo* to yield title compound as a pale yellow solid (3.0 g, 70% yield). ^1^H NMR (400 MHz, CDCl3) δ ppm 8.33 (d, *J* = 8.6 Hz, 2 H), 8.05 (s, 1 H), 7.30 (d, *J* = 8.6 Hz, 2 H), 2.87 (s, 3 H), 2.80 (s, 3 H), 2.40 (s, 3 H), 1.38 (s, 9 H). LC-MS (ESI): *m/z* 527; [M+H]^+^.

#### Step 7: General procedure for the synthesis of intermediates H1 through H4 via a palladium cross-coupling reaction

A degassed mixture of intermediate G (1equivalent), the corresponding boronic acid (1.2-1.5 equivalents), tetrakis(triphenylphosphine) palladium (0) (0.1 equivalents) and sodium carbonate (2 M aq, 2-3 equivalents) in 1,4-dioxane (0.1 M) was purged with N2 and stirred at 130 °C for 3 hours. The mixture was cooled down to 25 °C, diluted with ethyl acetate (EtOAc) and filtered through a pad of celite. The organic solution was washed with aqueous solution of NaHCO3 and brine. The organic layer was dried over Na_2_SO_4_, filtered and the solvents evaporated *in vacuo*.

##### Intermediate H1

*N*-(5-(2-Methoxyphenyl)-4,6-dimethyl-7-tosyl-7*H*-pyrrolo[2,3-*d*]pyrimidin-2-yl)pivalamide. The title compound was prepared according to the general procedure for step 7 from (2-methoxyphenyl)boronic acid (9.92 g, 65.3 mmol). Purification by chromatography (silica; 0-60% EtOAc:cyclohexane) provided Intermediate H1 as a yellow solid (14.3 g, 65% yield).^1^H NMR (400 MHz, CDCl3) δ ppm 8.34 (d, *J* = 8.6 Hz, 2 H), 8.07 (m, 1 H), 7.41 (d, *J* = 7.6 Hz, 1 H), 7.33 (d, *J* = 8.6 Hz, 5 H), 7.14 (dd, *J* = 7.3, 1.8 Hz, 1 H), 7.02 (m, 1 H), 6.97 (d, *J* = 8.3 Hz, 1 H), 3.70 (s, 3 H), 2.49 (s, 3 H), 2.42 (s, 3 H), 2.13 (s, 3 H), 1.38 (s, 9 H).

##### Intermediate H2

*N*-(5-(4-Fluoro-2-methoxyphenyl)-4,6-dimethyl-7-tosyl-7*H*-pyrrolo[2,3-*d*]pyrimidin-2-yl)pivalamide. The title compound was prepared according to general procedure for step 7 from 4-fluoro-2-methoxyphenyl)boronic acid (1.55 g, 9.12 mmol). Purification by chromatography (silica; 0-50% EtOAc:cyclohexane) provided Intermediate H2 as a yellow solid (3.61 g, 91% yield). ^1^H NMR (400 MHz, CDCl3) δ ppm 8.38 (d, *J* = 8.3 Hz, 2 H), 8.07 (br s, 1 H), 7.36 (d, *J* = 8.3 Hz, 3 H), 7.12 (dd, *J* = 8.3, 6.8 Hz, 1 H), 6.78 (dd, *J* = 10.9, 2.5 Hz, 1 H), 6.75 (td, *J* = 8.3, 2.4 Hz, 1 H), 3.72 (s, 3 H), 2.51 (m, 3 H), 2.45 (s, 3 H), 2.15 (s, 3 H), 1.41 (s, 9 H).

##### Intermediate H3

*N*-(5-(4-Chloro-2-methoxyphenyl)-4,6-dimethyl-7-tosyl-7*H*-pyrrolo[2,3-*d*]pyrimidin-2-yl)pivalamide. The title compound was prepared according to the general procedure for step 7 from 4-chloro-2-methoxyphenyl)boronic acid (2.76 g, 14.82 mmol). Purification by chromatography (silica; 0-50% EtOAc:cyclohexane) provided Intermediate H3 as a yellow solid (5.9 g, 88%). ^1^H NMR (400 MHz, CDCl3) δ ppm 8.36 (d, *J* = 8.3 Hz, 2 H), 8.03 (br s, 1 H), 7.34 (d, *J* = 8.3 Hz, 2 H), 7.08 (d, *J* = 8.1 Hz, 1 H), 7.03 (dd, *J* = 8.3, 1.8 Hz, 1 H), 6.96 (d, *J* = 1.8 Hz, 1 H), 3.70 (s, 3 H), 2.48 (m, 4 H), 2.42 (s, 3 H), 2.13 (s, 3 H), 1.38 (s, 9 H).

##### Intermediate H4

*N*-(5-(2,4-Dimethoxyphenyl)-4,6-dimethyl-7*H*-pyrrolo[2,3-*d*]pyrimidin-2-yl)pivalamide. The title compound was prepared according to general procedure for step 7 from 2,4-dimethoxyphenyl)boronic acid (734 mg, 4.04 mmol). The crude material was taken on to the next step (Step 10) without further purification

#### Step 8: General procedure for the deprotection of intermediates H1 and H2

The appropriate intermediate (H1 or H2) in methanol (0.2 M) was treated with sodium hydroxide (10 equivalents, 2 M aqueous solution) and the resulting mixture was stirred overnight at 100°C. The reaction mixture was cooled to 25 °C and extracted with dichloromethane (DCM). The organic layer was dried over Na_2_SO_4_, filtered and solvent evaporated *in vacuo*.

##### Compound 1

5-(2-Methoxyphenyl)-4,6-dimethyl-7H-pyrrolo[2,3-*d*]pyrimidin-2-amine, hydrochloride. Intermediate H1(12.63 g, 25 mmol) was deprotected following procedure described for step 8. Purification by chromatography (silica; 0-10% MeOH:DCM) provided 5-(2-methoxyphenyl)-4,6-dimethyl-7*H*-pyrrolo[2,3-*d*]pyrimidin-2-amine as a pale yellow solid (4.2 g, 63%). The solid was dissolved in 1,4-dioxane (52.2 ml) and treated with hydrochloric acid (4 M in dioxane, 3.91 ml, 15.65 mmol). The mixture stirred at 25 °C for 30 minutes. The precipitate formed was filtered, washed with 1,4-dioxane and dried *in vacuo* to provide title compound as a white solid (4.5 g, 94%). ^1^H NMR (400 MHz, DMSO-*d*6) δ ppm 13.94 (br s, 1 H), 12.27 (s, 1 H), 7.48 (br s, 2 H), 7.43 (td, *J* = 7.9, 1.8 Hz, 1 H), 7.25 (dd, *J* = 7.4, 1.6 Hz, 1 H), 7.14 (d, *J* = 7.8 Hz, 1 H), 7.05 (td, *J* = 7.4, 0.8 Hz, 1 H), 3.75(s, 3 H), 2.22 (s, 3 H), 2.15 (s, 3 H). ^13^C NMR (DMSO-*d6*, 101 MHz) δ 158.1, 154.9, 153.2, 149.1, 136.2, 132.7, 130.1, 122.0, 121.0, 112.2, 111.4, 110.2, 56.0, 16.1, 12.0. LC-MS (ESI): *m/z* 269; [M + H^+^]. Purity was determined as > 98% by HPLC (210-400 nm scan). Retention time (Rt): 7.09 minutes. (Sunfire C18 3,5u 2.1 x 100 mm, TFA 0.1% in water/TFA 0.1% in ACN, 50 °C).

##### Compound 2

5-(4-Fluoro-2-methoxyphenyl)-4,6-dimethyl-7*H*-pyrrolo[2,3-*d*]pyrimidin-2-amine. Intermediate H2 (350mg, 0.95mmol) was deprotected following general procedure described in step 8. The crude residue obtained after evaporation of extraction solvents was triturated with EtOAc and cyclohexane to provide title compound as a beige solid (140 mg, 52%).^1^H NMR (400 MHz, DMSO-*d*6) δ ppm 11.00 (s, 1 H), 7.18 (dd, *J* = 8.2, 7.2 Hz, 1 H), 6.97 (dd, *J* = 11.5, 2.4 Hz, 1 H), 6.80 (td, *J* = 8.3, 2.5 Hz, 1 H), 5.83 (br s, 2 H), 3.71 (s, 2 H), 2.04 (s, 3 H), 1.96 (s, 3 H). LC-MS (ESI): *m/z* 287; [M + H^+^]. Purity was determined as > 95% by HPLC (320 nm). Retention time (Rt): 1.05 minutes. (Acquity UPLC BEH C18).

#### Step 10: General procedure for the deprotection of intermediates H3 and H4

*Tosyl group deprotection*. Sodium hydroxide (20-30 equivalents) was added to a stirred solution of intermediate H3 or H4 in Methanol (0.03-0.05 M) and the mixture was stirred at room temperature (16-72 hours). Reaction solvents were removed, and the resulting mixture was extracted with 10% MeOH/DCM three times. The organic layer was dried over Na_2_SO_4_, filtered and the solvents evaporated *in vacuo*. Residue was used in the next deprotection step without any further purification.

*Pivaloyl deprotection*. Sodium hydroxide (7 equivalents) was added to a stirred solution of crudes obtained from previous step in Methanol (0.1 M) and the mixture was stirred for at 90 °C (4-16 hours). The mixture was cooled down and was extracted with DCM three times. The organic layer was dried over Na_2_SO_4_, filtered and the solvents evaporated *in vacuo*.

The mixture was cooled down to room temperature and extracted with DCM three times. The organic layer was dried over Na_2_SO_4_, filtered and the solvents evaporated *in vacuo*.

##### Compound 3

5-(4-Chloro-2-methoxyphenyl)-4,6-dimethyl-7*H*-pyrrolo[2,3-*d*]pyrimidin-2-amine. Intermediate H3 (5.9g, 11 mmol) was deprotected following general procedure for Step 10. Purification by chromatography (silica; 50% Cy-3:1 EtOH:EtOAc) provided title compound as a white solid (3 g, 91% yield over 2 steps).^1^H NMR (400 MHz, DMSO-*d*6) δ ppm 11.02 (s, 1 H), 7.18 (d, *J* = 8.1 Hz, 1 H), 7.12 (d, *J* = 2.0 Hz, 1 H), 7.04 (dd, *J* = 8.1, 2.0 Hz, 1 H), 5.83 (br s, 2 H), 3.72 (s, 3 H), 2.05 (s, 3 H), 1.97 (s, 3 H, CH3); LC-MS (ESI): *m/z* 303, 305; [M + H^+^], 1x Cl isotopic pattern observed. Purity was determined as > 98% by HPLC (summed signal from 210 to 600 nm). Retention time (Rt): 1.11 minutes. (Acquity UPLC BEH C18).

##### Compound 4

5-(2,4-Dimethoxyphenyl)-4,6-dimethyl-7*H*-pyrrolo[2,3-*d*]pyrimidin-2-amine. Intermediate H4 (1.69 g, 2.5 mmol) was deprotected following general procedure for Step 10. Part of the reaction solvent were removed under vacuum and the remaining mixture was diluted with water and extracted with 10% MeOH:DCM three times. The combined organic layers were dried over Na_2_SO_4_, filtered and solvents evaporated *in vacuo*. Resulting residue was purified by chromatography (silica; 0-10% MeOH:DCM) providing title compound as a white solid (760 mg, quantitative yield).

^1^H NMR (400 MHz, DMSO-*d*6) δ ppm 10.92 (s, 1 H), 7.05 (d, *J* = 8.1 Hz, 1 H), 6.61 (d, *J* = 2.5 Hz, 1 H), 6.56 (dd, *J* = 8.2, 2.4 Hz, 1 H), 5.79 (br s, 2 H), 3.80 (s, 3 H), 3.67 (s, 3 H), 2.03 (s, 3 H), 1.97 (s, 3 H); LC-MS (ESI): *m/z* 299; [M + H^+^]. Purity was determined as > 99% by HPLC (summed signal from 210 to 600 nm). Retention time (Rt): 2.23 minutes. (Acquity UPLC BEH C18).

##### Synthesis of compound 5

###### Intermediate I

*N*-(5-(4-Fluoro-2-methoxyphenyl)-4,6-dimethyl-7*H*-pyrrolo[2,3-*d*]pyrimidin-2-yl)pivalamide. A stirred solution of intermediate H2 (1.02 g, 1.9 mmol) in methanol (5 ml) was treated with sodium hydroxide (2 M, 4.9 ml, 9.7 mmol) and the resulting mixture stirred at room temperature overnight. The reaction mixture was extracted with DCM and the organic layer was dried over sodium sulfate, filtered and solvents removed *in vacuo*. The residue was purified by flash column chromatography (silica; 0-10% DCM:MeOH). Fractions containing product were combined and the solvents evaporated *in vacuo*. The resulting residue was triturated with diethyl ether to provide title compound as a beige solid (57 6mg, 80% yield). ^1^H NMR (400 MHz, DMSO-*d*6) δ ppm 8.95 (br s, 1 H), 8.06 (br s, 1H), 7.16 (m, 1H), 6.74 (m, 2H), 3.74 (s, 3 H), 2.24 (s, 3 H), 2.23 (s, 3 H), 1.36 (s, 9 H).

###### Compound 5

5-(4-Fluoro-2-methoxyphenyl)-4,6,7-trimethyl-7*H*-pyrrolo[2,3-*d*]pyrimidin-2-amine. A solution of iodomethane (0.336 ml, 5.40 mmol) in DMF (6 ml) was added to a stirred solution of intermediate I (400 mg, 1.080 mmol) in DMF (6 ml) and the resulting mixture stirred at 25 °C overnight. The reaction mixture was diluted with water and extracted with EtOAc three times. The combined organic layers were dried over Na_2_SO_4_, filtered and the solvents evaporated *in vacuo*. The resulting yellow oil was used in the next step without further purification. The crude material was dissolved in methanol (10 ml) and treated with sodium hydroxide (2 M, 10.4 ml, 21 mmol) and the mixture stirred at 100 °C overnight. The mixture was cooled down to room temperature and extracted with DCM. The organic layer was dried over Na_2_SO_4_, filtered and solvents evaporated *in vacuo*. Residue was purified by chromatography (silica; 0-10% MeOH:DCM) to provide title compound as a beige solid (100 mg, 33%). ^1^H NMR (400 MHz, DMSO-*d*_6_) δ ppm 7.17 (dd, *J* = 8.2, 7.2 Hz, 1 H), 6.98 (dd, *J* = 11.6, 2.5 Hz, 1 H), 6.82 (td, *J* = 8.4, 2.7 Hz, 1 H), 5.98 (s, 2 H), 3.71 (s, 3 H), 3.54 (s, 3 H), 2.08 (s, 3 H), 1.96 (s, 3 H). LC-MS (ESI): *m/z* 301; [M + H^+^]. Purity was determined as > 95% by HPLC (240 nm). Retention time (Rt): 1.11 minutes. (Acquity UPLC BEH C18).

## Supplementary Material

Supplementary Materials

## Figures and Tables

**Table 1:** *in vitro* activities of pyrrolopyrimidine analogues against *T. cruzi*

Compound		Benznidazole	Posaconazole	1	2	3	4
**Amastigotes**	**pEC** **_50_**	5.7 ± 0.2	8.6 ± 0.1	6.5± 0.1	7.3 ± 0.2	8.0 ± 0.1	7.1 ± 0.2
	**EC** **_50_**	2.0 μM	0.003 μM	0.32 μM	0.05 μM	0.01 μM	0.08 μM
**Vero**	**pEC** **_50_**	< 4.3	< 4.3	< 4.3	< 4.3	< 4.3	< 4.3
	**EC** **_50_**	>50 μM	>50 μM	>50 μM	>50 μM	>50 μM	>50 μM
**Trypomastigotes**	**pEC** **_50_**	5.4 ± 0.03	< 4.3	6.6 ± 0.3	7.5 ± 0.1	7.9 ± 0.1	7.3 ± 0.1
	**EC** **_50_**	4.0 μM	>50 μM	0.25 μM	0.03 μM	0.01 μM	0.05 μM
**CYP51**	**PIC** **_50_**	ND	7.3 ± 0.1	< 4	< 4	< 4	< 4
	**IC** **_50_**		0.05 μM	>100 μM	>100 μM	>100 μM	>100 μM
**HepG2**	**pEC** **_50_**	<4	<4	<4	4.2 ± 0.2	4.1 ± 0.1	4.1 ± 0.1
	**EC** **_50_**	>100 μM	>100 μM	>100 μM	79 μM	79 μM	79 μM
**Chrom log*D* pH 7.4**		2.3	6.1	3.2	3.4	4.2	3.1
**PFI**		4.3	11.1	6.2	6.4	7.2	6.1
**Solubility pH 7.4 (** **p** **g/ml)**		77	14	110	117	103	137
**FaSSIF pH 6.5 (** **p** **g/ml)**		ND	ND	456	153	76	107
**Cl** **_int_** **mouse/human** **(ml/min/g liver)**		ND	ND	ND	1.3/0.6	1.4/0.4	2.0/0.8
**T** **_1/2_** **mouse/human** **(min)**		ND	ND	ND	52/96	48/>139	33/66
**Mouse PPB %*^e^***		ND	ND	ND	82	97	78

All compound structures and reaction schemes shown in Fig. 1, Fig. S1 and S2. All data shown as pEC_50_/pIC_50_ ± standard deviation and mean EC_50_/IC_50_ (= 10^-pEC_50_^ × 10^6^). Results are from a minimum of three independent experiments, as described in Materials and Methods. Amastigotes: intracellular *T. cruzi* amastigotes, strain Silvio X10/7 A1; VERO: cytotoxicity against host cells in intracellular amastigote assay; trypomastigotes: *T. cruzi* culture derived trypomastigotes, strain Silvio X10/7 A1; CYP51: *T. cruzi* CYP51 biochemical assay; HepG2: potency against mammalian HepG2 cells; PFI is Property Forecast Index (number of aromatic rings + ChromLogD at pH 7.4); Kinetic aqueous solubility at pH 7.4 measured by chemiluminescent nitrogen detection; FaSSIF solubility is Fasted State Simulated Intestinal Fluid solubility; Cl_int_ is intrinsic clearance ; T_1/2_ is half-life; PPB refers to plasma protein binding; ND, not determined

**Table 2:** Assessment of compound efficacy and measurement of complex III activity.

Compound	EC_50_ (nM) values for WT and resistant lines (fold change from WT)			Complex III assay IC_50_ (nM)
	WT	L197I	F222L	
2	33 ± 2	117 ± 5 (4)	3620 ± 322 (109)	8 ± 1
3	6 ± 0.2	115 ± 7 (19)	1120±63 (183)	2 ± 0.3
4	88 ± 3	432 ± 15 (5)	6380 ± 436 (73)	6 ± 1
5	2 ± 0.1	21 ± 0.5 (10)	486 ± 18 (224)	1 ± 0.2

EC_50_ and IC_50_ values against *T. cruzi* epimastigotes of wild-type (WT), resistant lines of compound 3 (L197I) and compound 5 (F222L) and inhibition of complex III activity (epimastigote lysates). EC_50_ values are the mean of at least two biological replicates consisting of at least two technical replicates (n ≥ 2).

**Table 3:** Assessment of human complex III activity and mitochondrial toxicity.

Assay	Compounds	
	3	4
Human complex III EC_50_ (μM)	20% inhibition at 200μM	>200
Calcium loading capacity, rat mitochondria, MEC (μM)	5.7	>200
Seahorse MST, HepG2, LEC (μM)	80	>80

Human complex III EC_50_ values are the mean of at least two biological replicates consisting of at least two technical replicates (n ≥ 2). For dose response data see Tables S6 and S7.

MEC = Minimum Effective Concentration, was defined as the lowest concentration where the fitted cubic polynomial regression curve exceeded three standard deviations around the DMSO mean response.

LEC = Lowest Effective Concentration for the mitochondrial stress test in HepG2 cells, was estimated for each compound based on the test concentration causing a reduction in oxygen consumption rate >15% compared to DMSO control in at least two out of three replicates.

## Data Availability

All data presented in this manuscript is available in the paper, supplementary files or deposited on Dryad (https://doi.org/10.5061/dryad.95x69p8r7). Compounds will be provided by contacting T.J.M. and subject to an appropriate MTA being put in place and compound availability.

## One-sentence summary

A five-day combination regimen of a cytochrome *b* inhibitor and a low dose of benznidazole prevents relapse in an experimental model of chronic Chagas disease.
