# Supplementary material for: Short-course combination treatment for experimental chronic Chagas disease
Source: Sci Transl Med. Author manuscript; Available in PMC 2024 Feb 29. (PMC7615676; doi:10.1126/scitranslmed.adg8105)
Supplement: Supplementary Materials [file EMS194243-supplement-Supplementary_Materials.pdf]

Supplementary Materials for

Short-course combination treatment for experimental chronic Chagas disease.

Authors: Silvia González[1], Richard J. Wall[5#], John Thomas[5], Stephanie Braillard[6], Gino Brunori[2], Isabel Camino Díaz[3], Juan Cantizani[1], Sandra Carvalho[5], Pablo Castañeda Casado[3], Eric Chatelain[6], Ignacio Cotillo[1], Jose M. Fiandor[1], Amanda Fortes Francisco[4], David Grimsditch[2], Martine Keenan[7‗], John M. Kelly[4], Albane Kessler[1‡], Chiara Luise[5¬], Jon J. Lyon[2], Lorna MacLean[5], Maria Marco[1], J. Julio Martin[1‖], Maria S. Martinez Martinez[3], Christy Paterson[5], Kevin D. Read[5], Angel Santos-Villarejo[3], Fabio Zuccotto[5~], Susan Wyllie[5*], Tim J. Miles[1*] and Manu De Rycker[5*]

* corresponding authors

Susan Wyllie: s.wyllie@dundee.ac.uk

Tim Miles: tim.j.miles@gsk.com

Manu De Rycker: m.derycker@dundee.ac.uk

This file includes:

Materials and Methods

Fig. S1. Synthesis of compounds 1,2,3, and 4.

Fig. S2. Synthesis of compound 5 used in mode of action studies

Fig. S3. Panel of *T. cruzi* strains.

Fig. S4. Rate-of-kill determination for pyrrolopyrimidine compounds.

Fig. S5. 16-day washout experiment and potency profiling of recrudescent trypomastigotes.

Fig. S6. *In vitro* potency interaction screening between benznidazole and Compounds 2 and 3.

Fig. S7. *In vitro* benznidazole – posaconazole washout combination study

Fig. S8. Combination rate-of-kill data.

Fig. S9. 2D ligand-protein interaction diagrams of the predicted binding modes of compounds 1-4 in the Qi site of *T. cruzi* cytochrome *b*.

Fig. S10. X-ray binding mode of antimycin A and predicted binding mode of compound 3.

Fig. S11. Ligand-protein interaction diagram of 100 ns MD simulation of compound 3 in complex with *T. cruzi* cytochrome *b*

Fig. S12. Examples of binding modes during 100 ns MD simulation of compound 3 in complex with *T. cruzi* cytochrome *b*

Fig. S13. Summary of 100 ns MD simulation of compound 3 in complex with *T. cruzi* cytochrome *b*.

Fig. S14 Analysis of resistance mutations in Q_i site of *T. cruzi* cytochrome *b*.

Fig. S15. Predicted binding modes of compounds 1-4 in the *T. cruzi* cytochrome *b* pocket bearing L197I mutation.

Fig. S16. 2D ligand-protein interaction diagrams of the predicted binding modes of compounds 1-4 in the *T. cruzi* cytochrome *b* pocket bearing L197I mutation.

Fig. S17. Predicted binding modes of compound 3 in the *T. cruzi* cytochrome *b* pocket bearing F222L mutation.

Fig. S18. Washout outgrowth assay for compound 4.

Table S1. Efficacy of compounds 2 and 3 against chronic *T. cruzi* infections with different dosing regimens and comparison with BNZ.

Table S2. Bioanalysis of samples from *in vivo* efficacy studies with compounds 2 and 3 against chronic *T. cruzi* infections with different dosing regimens.

Table S3. Bioanalysis of samples from *in vivo* efficacy studies with compounds 3 co-administered with BNZ for 5 days in a chronic *T. cruzi* mouse infection.

Table S4. Whole genome sequencing overview, read counts and coverage.

Table S5. Bioanalysis of samples from *in vivo* efficacy studies with compounds 4 co-administered with BNZ for 5 days in a chronic *T. cruzi* mouse infection.

Table S6. Percentage inhibition of human bc1 at various concentrations of compound 3.

Table S7. Percentage inhibition of human bc1 at various concentrations of compound 4.

Other Supplementary Materials for this manuscript are available on DRYAD:
https://doi.org/10.5061/dryad.95x69p8r7

Supplementary figures

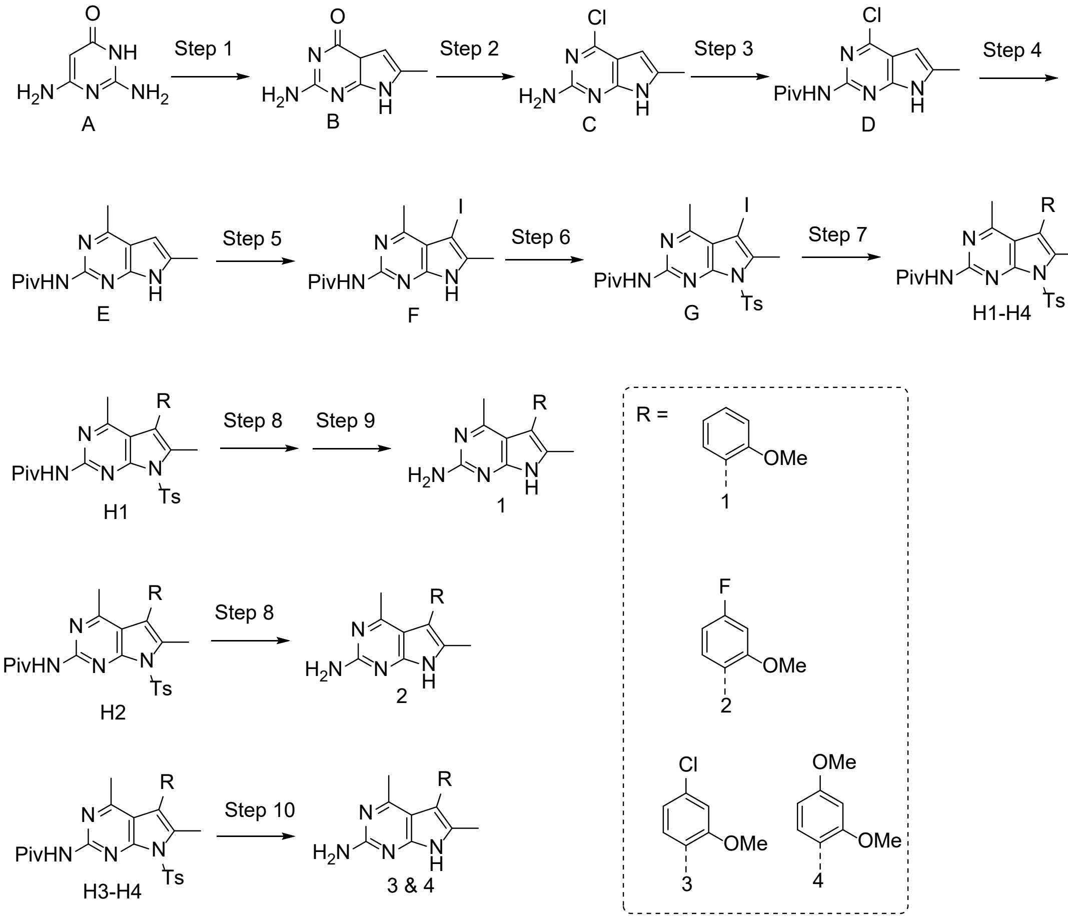

Fig. S1. Synthesis of compounds 1,2,3, and 4.

Reagents and conditions: Step 1, Sodium acetate, chloroacetone, H_2O, reflux, 5 h; Step 2, Phosphorus oxychloride, $PhNMe_2$, 110 ºC, 3 h; Step 3, Pivaloyl chloride, pyridine, 25 ºC, 3 h; Step 4, 2M trimethylaluminium, $Pd(PPh_3)_4$, THF, 75 ºC, 16 h; Step 5, NIS, THF, 25 ºC, 2h; Step 6, Sodium hydride, 4-methylbenzenesulfonyl chloride, THF, 0 -25ºC, 16 h (71%); Step 7, boronic acid, sodium carbonate, $Pd(PPh_3)_4$, dioxane, 130 ºC, 3 h; Step 8, 2M Sodium hydroxide, methanol, 100 ºC, 16 h; Step 9, 4 M HCl, dioxane, 25 ºC, 30 min; Step 10, i) 2 M Sodium hydroxide, methanol, 25 ºC, 16 h; ii) 2 M Sodium hydroxide, methanol, 100 ºC, 16 h

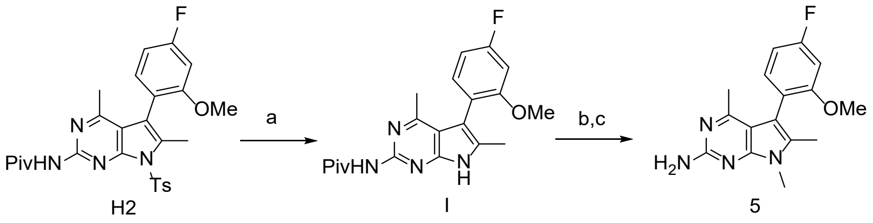

Fig. S2. Synthesis of compound 5 used in mode of action studies.

Reagents and conditions: a) 2 M Sodium hydroxide, methanol, 25 ºC, 16 h; b) Cs_2CO_3, MeI, DMF, 25 ºC, 16 h; c) 2 M Sodium hydroxide, methanol, 100 ºC, 16 h.

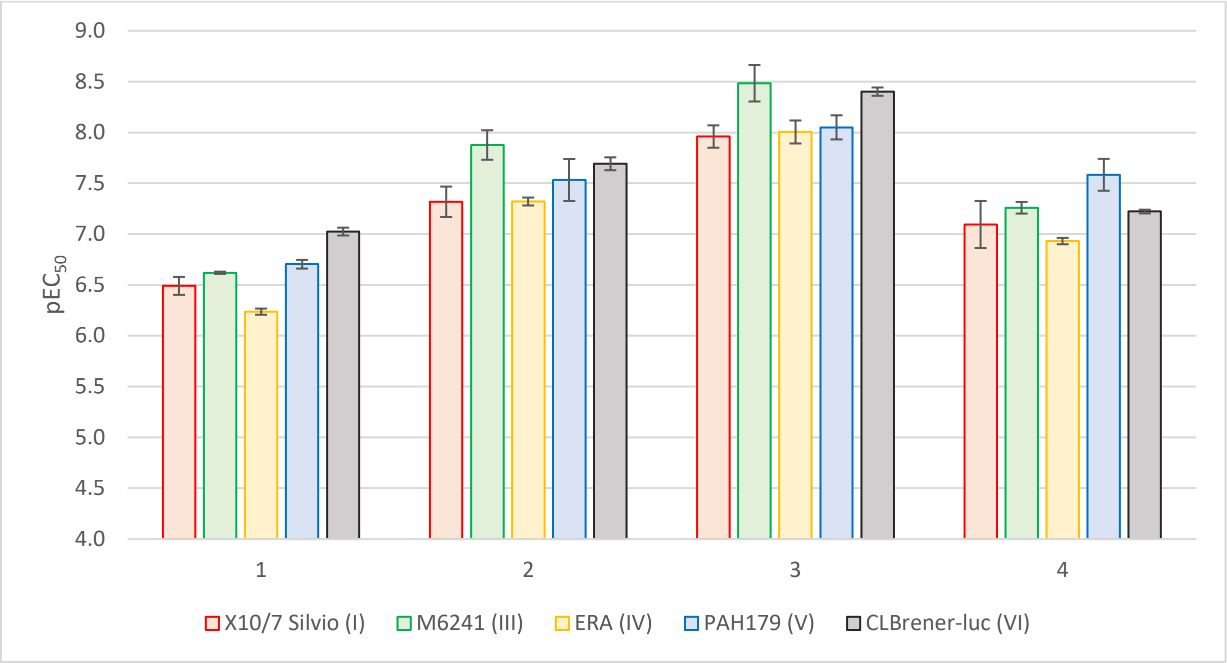

Fig. S3. Panel of *T. cruzi* strains. Key compounds were tested against strains from multiple discrete typing units (DTU, shown in brackets for each strain). pEC_{50} reported is average of at least three biological replicates. Error bars represent standard deviation.

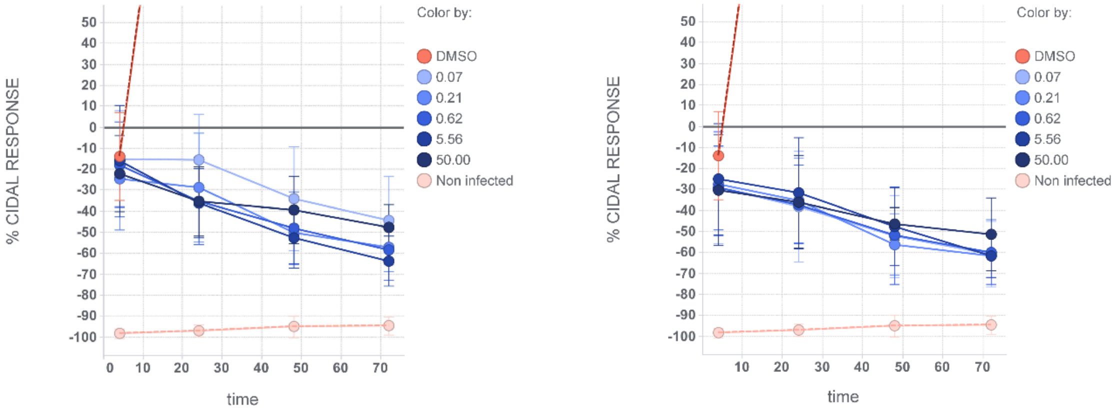

Fig. S4. Rate-of-kill determination for pyrrolopyrimidine compounds. Determination of rate-of-kill against intracellular amastigotes for compounds 2 (left) and 3 (right). Experiments were carried out as described in (*38*). Data is normalized to starting average number of intracellular parasites at t = 4 h post plating (0% cidal response) and average number of intracellular amastigotes in un-infected cells at each timepoint (100% cidal response) and is represented as mean +/- standard deviation based on at least 5 independent replicates.

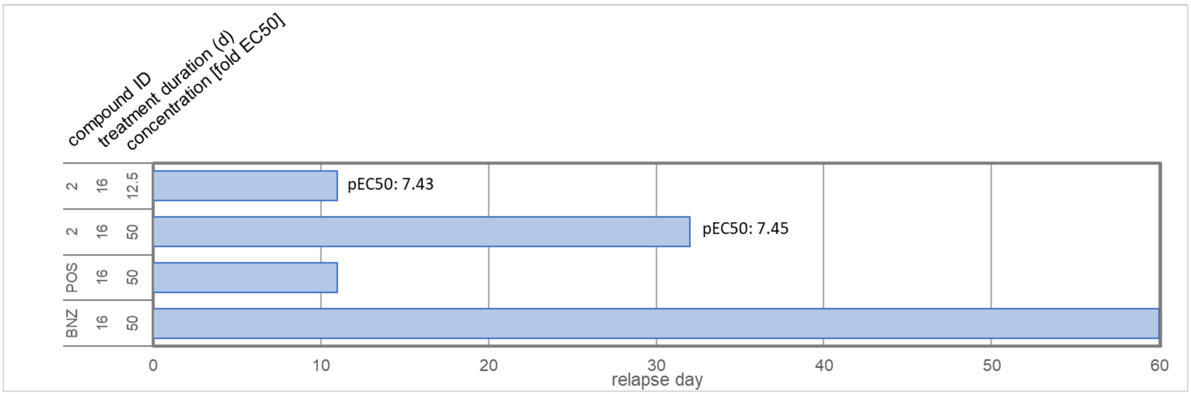

Fig. S5. 16-day washout experiment and potency profiling of recrudescent trypomastigotes. Intracellular parasites were subjected to treatment with compounds 2, posaconazole (POS) and BNZ at the indicated concentrations and for 16 days (with compounds replenished every four days), followed by extensive washout of compounds and assessment of parasite relapse for 60 days post washout. Relapse day indicates the first day after washout that viable parasites were observed microscopically. pEC_{50} = potency of compound 2 against trypomastigotes recovered after relapse, compare with pEC_{50} of 7.5 for untreated trypomastigotes (Table 1).

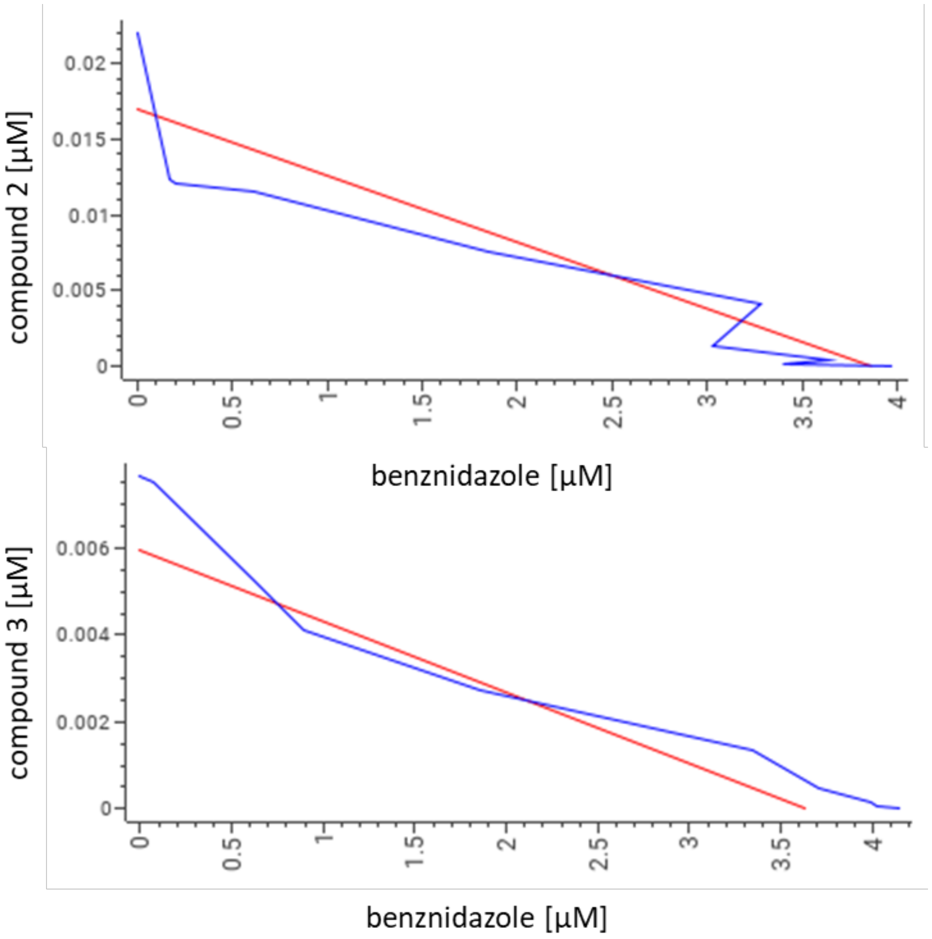

Fig. S6. *In vitro* potency interaction screening between benznidazole and compounds 2 and 3.

Interaction analysis of compound 2 and benznidazole (top panel) and compound 3 and benznidazole (bottom panel). For both combination pairs a 11x8 dose response matrix experiment was carried out in duplicate. Isobolograms were calculated using Genedata Screener. A good fit was obtained in both cases (reduced chi-square 5.1 and 4.1 respectively). Red line shows theoretical line of additivity, blue line shows data for compound pair. The median combination index for compound 2 + benznidazole is 0.66 (range 0.56 – 0.81). The median combination index for compound 3 + benznidazole is 0.69 (range 0.44 – 0.78). In both cases this indicates an additive interaction at the potency level.

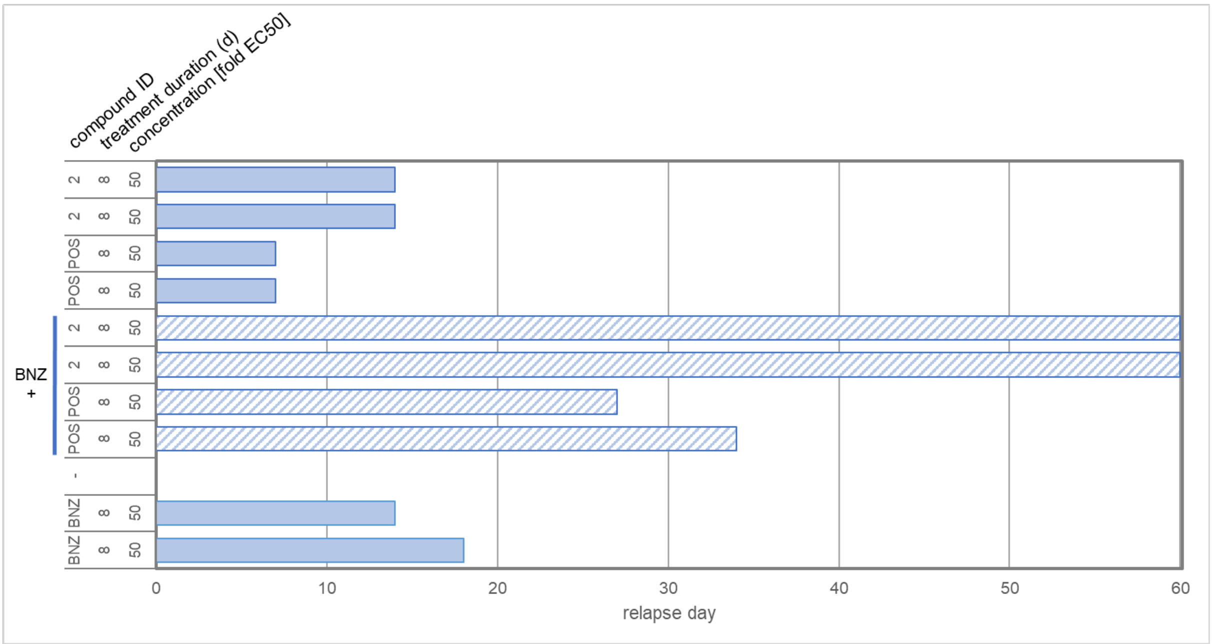

Fig. S7. *In vitro* benznidazole – posaconazole washout combination study

Washout outgrowth assay with posaconazole (POS), compound 2 and BNZ. The fixed concentration of BNZ used in the combinations (hashed bars) was 12.5× the established EC_{50} value. Relapse day is first day post washout of compounds when viable parasites were observed. Two technical replicates for each condition.

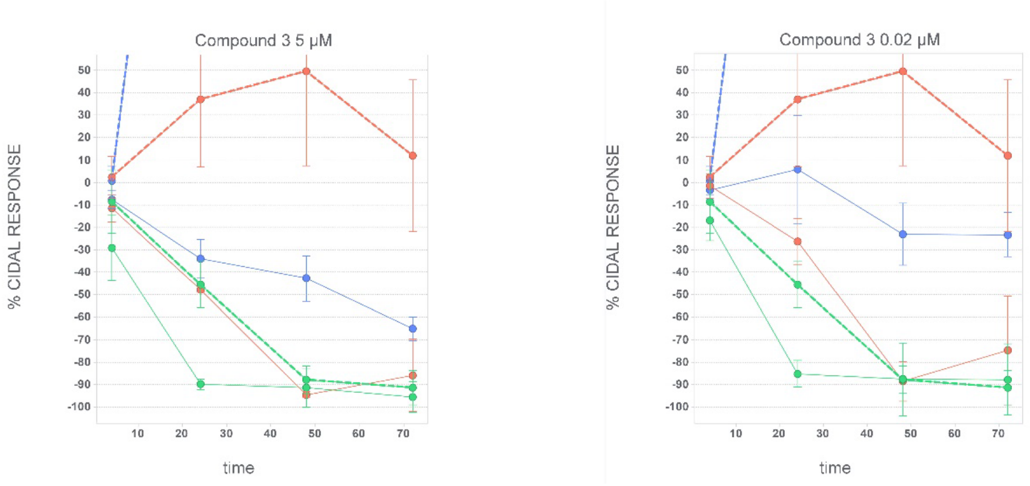

Fig. S8. Combination rate-of-kill data

Determination of rate-of-kill against intracellular amastigotes for compound 3 in presence or absence of benznidazole. Left panel: 5 µM compound 3 was tested in absence or presence of benznidazole (solid lines, blue = 0 µM benznidazole, red = 10 µM benznidazole, green = 50 µM benznidazole). Dashed lines show data for benznidazole-only treatment. Right panel: same as left, but with 0.02 µM compound 3. Data is normalized to starting average number of intracellular parasites at t = 4 hours post plating (0% cidal response) and average number of intracellular amastigotes

in un-infected cells at each timepoint (-100% cidal response). Data is from three independent replicates, error bars represent standard deviation.

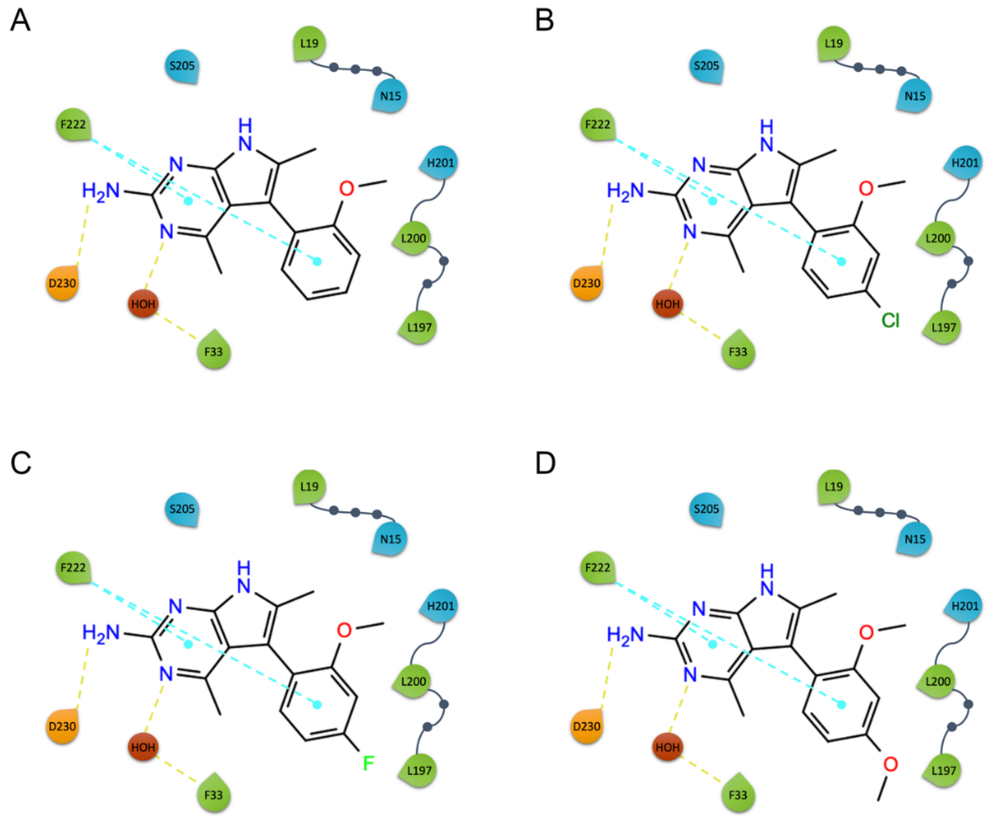

Fig. S9. 2D ligand-protein interaction diagrams of the predicted binding modes of compounds 1-4 at the *T. cruzi* cytochrome *b* pocket.

(A) Compound 1. (B) Compound 3. (C) Compound 2. (D) Compound 4. Binding interactions are represented with dashed lines colored in yellow (hydrogen bond) and cyan (π-π stacking).

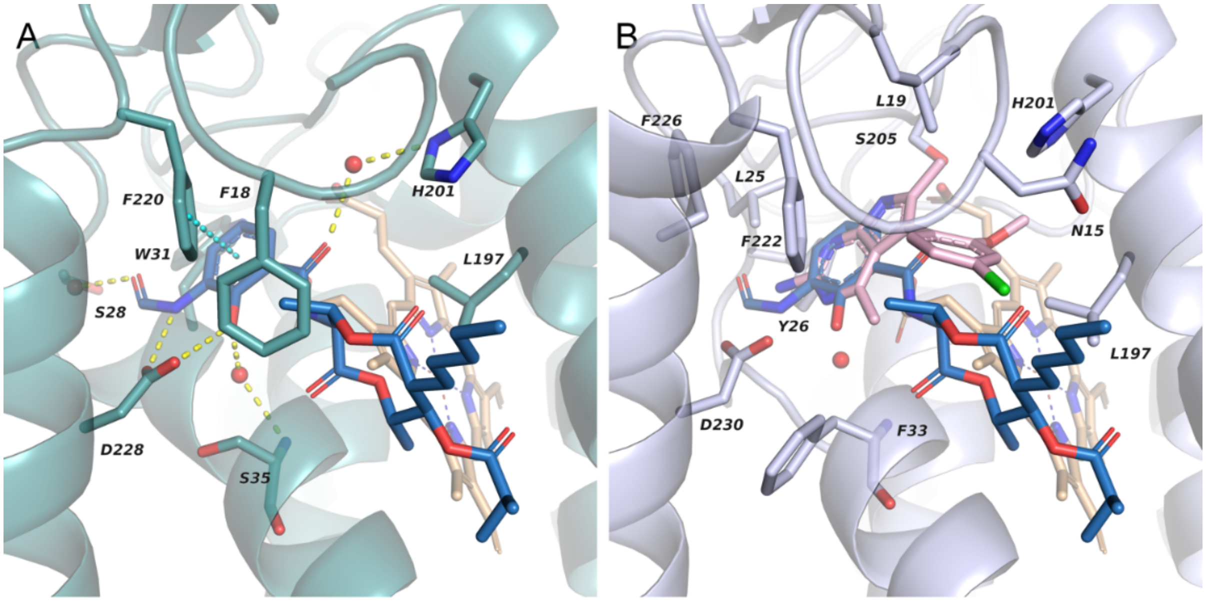

Fig. S10. X-ray binding mode of antimycin A and predicted binding mode of compound

Compound 3 in the Q_i site of cytochrome *b*. (**A**) Crystal structure of antimycin A in the Q_i site of the bc_1 complex (PDB ID: 1PPJ). (**B**) Predicted binding mode of compound compound 3 (pink) at the T. *cruzi* cytochrome *b* pocket (light grey) superimposed with the experimentally determined binding mode of antimycin A (blue; PDB ID: 1PPJ). For clarity, only the side chains of the surrounding amino acid residues are shown as sticks; for residues S35 and F33, the backbone atoms are also shown. The heme group is depicted as beige sticks, while the oxygen atoms of the water molecules as red spheres. Binding interactions are represented with dashed lines coloured in yellow (hydrogen bond) and cyan (π-π stacking).

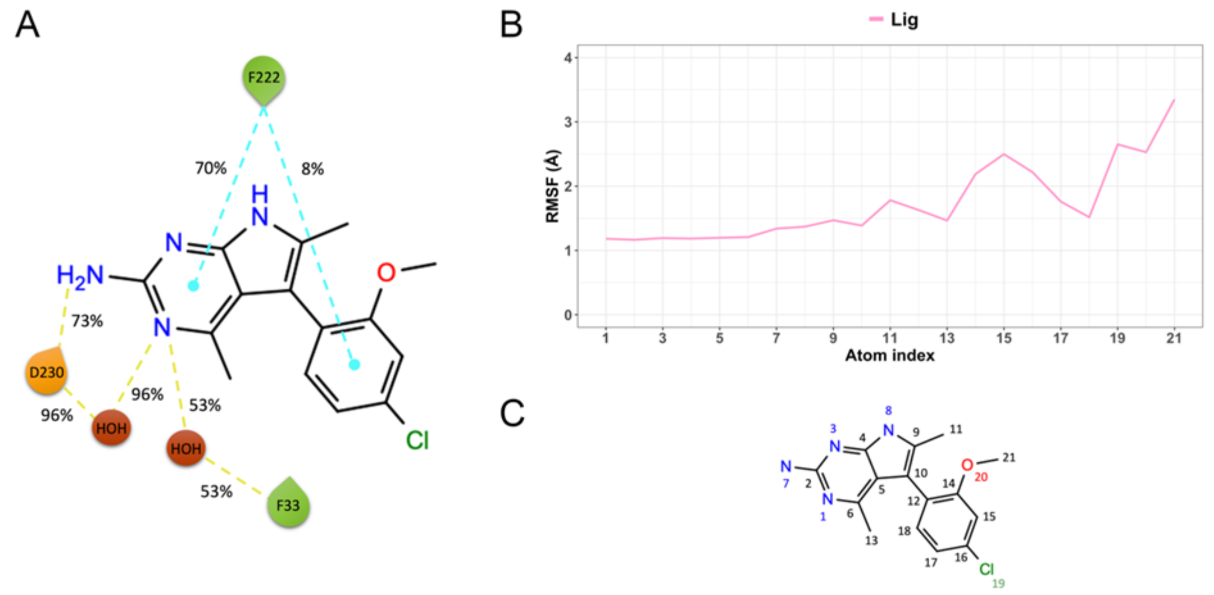

Fig. S11. Ligand-protein interaction diagram of 100 ns MD simulation of compound 3 in complex with *T. cruzi* cytochrome *b*.

(**A**) 2D ligand-protein interaction diagram with the interaction occurrences. Binding interactions are represented with dashed lines coloured in yellow (hydrogen bond) and cyan (π-π stacking) (**B**) Ligand root mean square fluctuations (RMSF) values broken down by atom. (**C**) 2D structure of compound 3 with the corresponding atom index for the ligand's fluctuations.

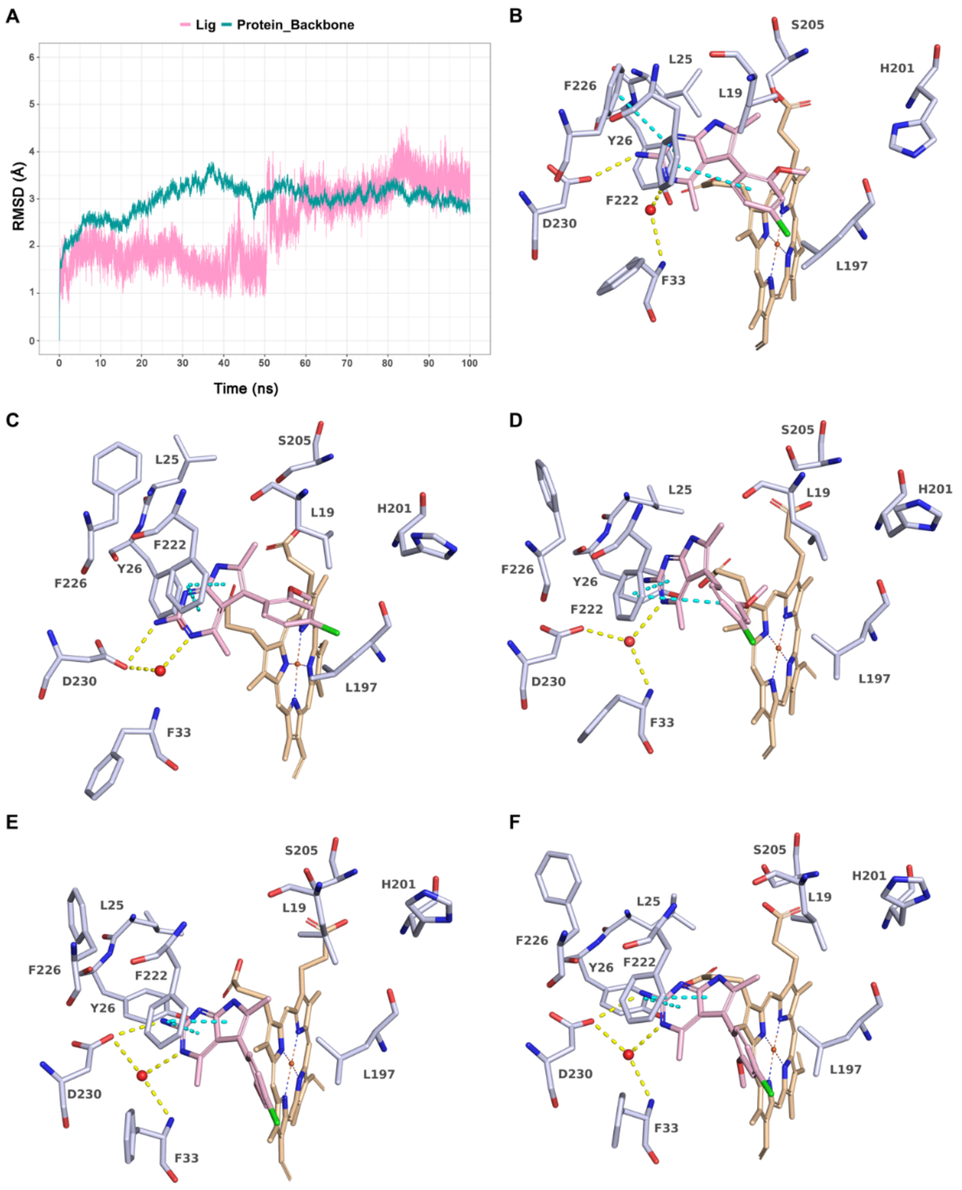

Fig. S12. Examples of binding modes during 100 ns MD simulation of compound 3 in complex with *T. cruzi* cytochrome *b*.

(**A**) Root mean square deviation (RMSD) values of the protein (backbone atoms) and ligand (heavy atoms). Binding mode of compound 3 observed during the MD simulation at 0 ns (**B**), 25 ns (**C**), 50 ns (**D**), 75 ns (**E**), and 100 ns (**F**). Only the surrounding amino acid residues are shown for clarity, and they are displayed as light grey sticks. The heme group is depicted as beige sticks, while the oxygen atom of the water molecule as a red sphere. Binding interactions are represented with dashed lines colored in yellow (hydrogen bond) and cyan (π-π stacking).

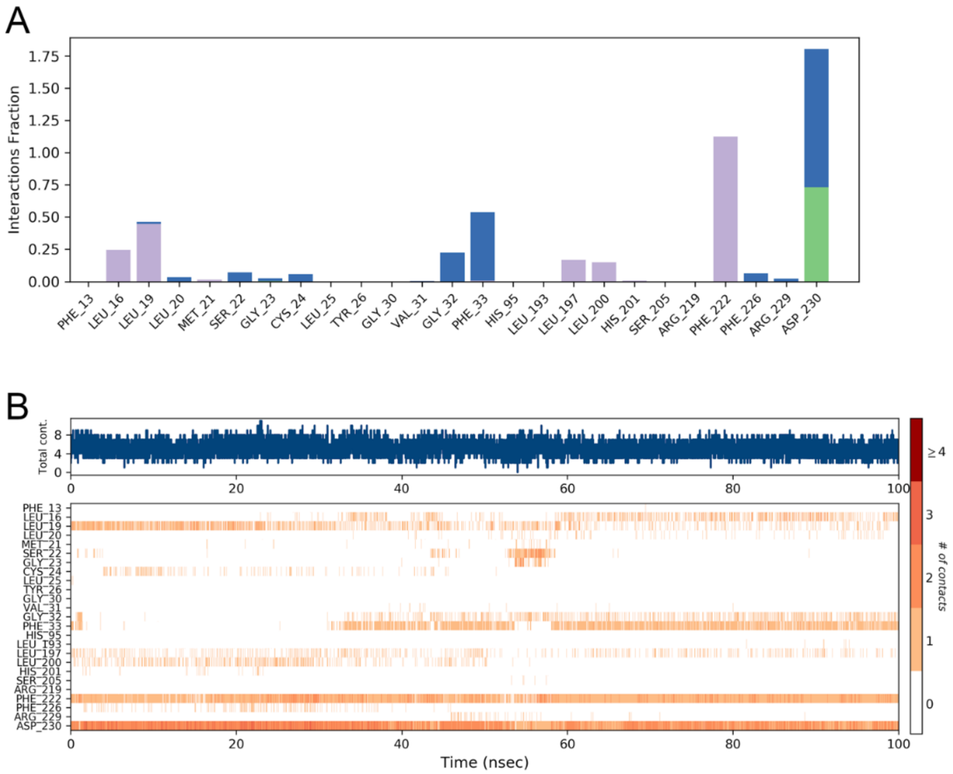

Fig. S13. Summary of 100 ns MD simulation of compound 3 in complex with *T. cruzi* cytochrome *b*.

(**A**) Summary of the protein-ligand contacts during the MD simulation. Binding interactions colors: green (hydrogen bond), lilac (hydrophobic), blue (water bridges). Values over 1.0 indicate that the protein residues make multiple contacts with the ligand. (**B**) Timeline representation of the interactions and contacts (hydrogen bond, hydrophobic, water bridges). The top panel shows the total number of specific protein-ligand contacts over the course of the trajectory. The bottom panel displays which residues interact with the ligand in each trajectory frame.

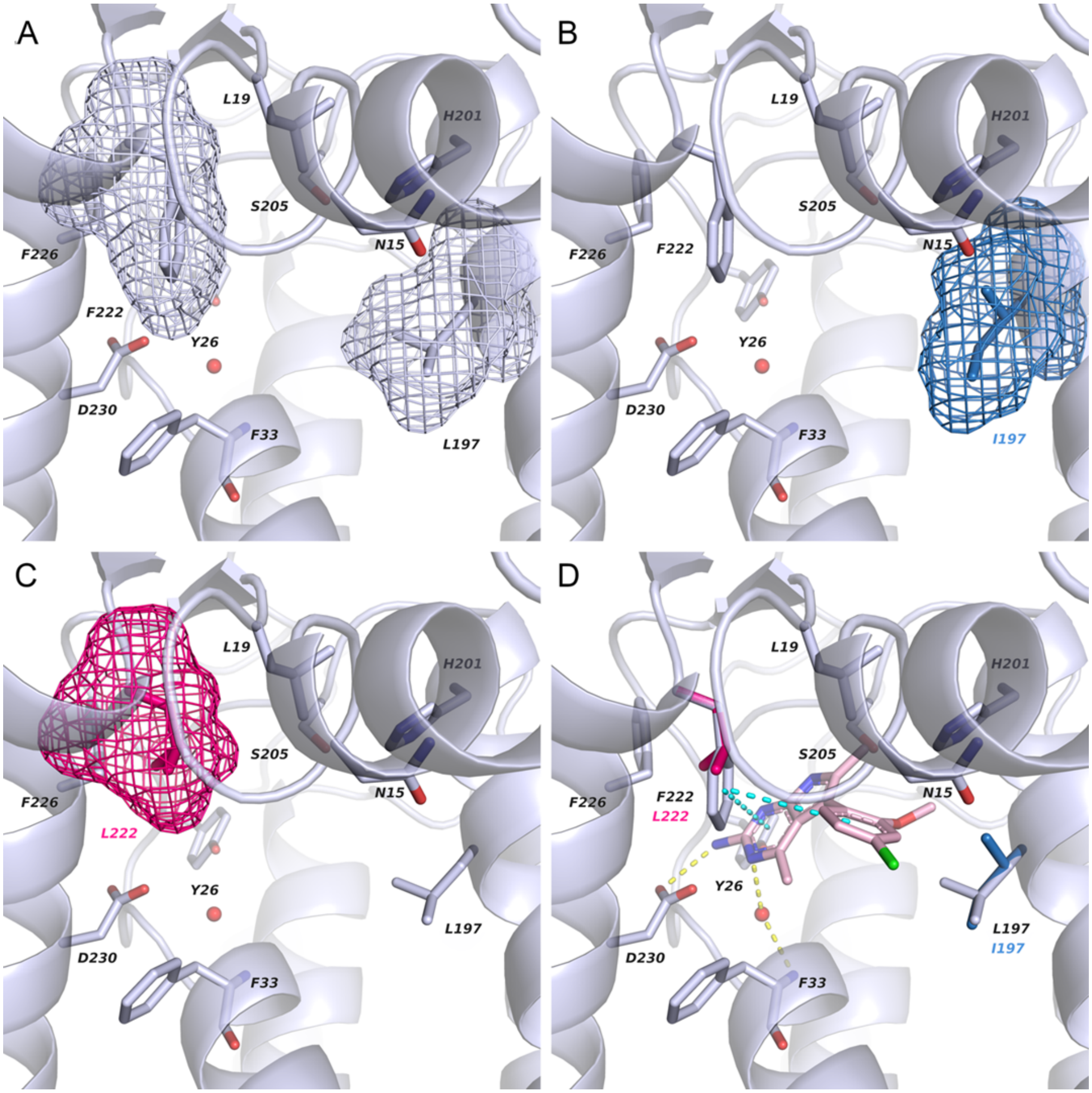

Fig. S14 Analysis of resistance mutations in Q$_i$ site of *T. cruzi* cytochrome *b*.

(**A**) WT. (**B**) L197I mutation. (**C**) F222L mutation. (**D**) Predicted binding mode of compound 3 (pink) at the *T. cruzi* cytochrome *b* pocket (light grey) superimposed with I197 (blue) and L222 (magenta) mutations. Only the side chain residues are shown for clarity, and they are displayed as sticks; for residue F33, the main chain is shown. L197, I197, F222 and L222 are also illustrated as surface mesh. Binding interactions are represented with dashed lines colored in yellow (hydrogen bond) and cyan (π-π stacking), while the conserved water molecule is depicted as a red sphere.

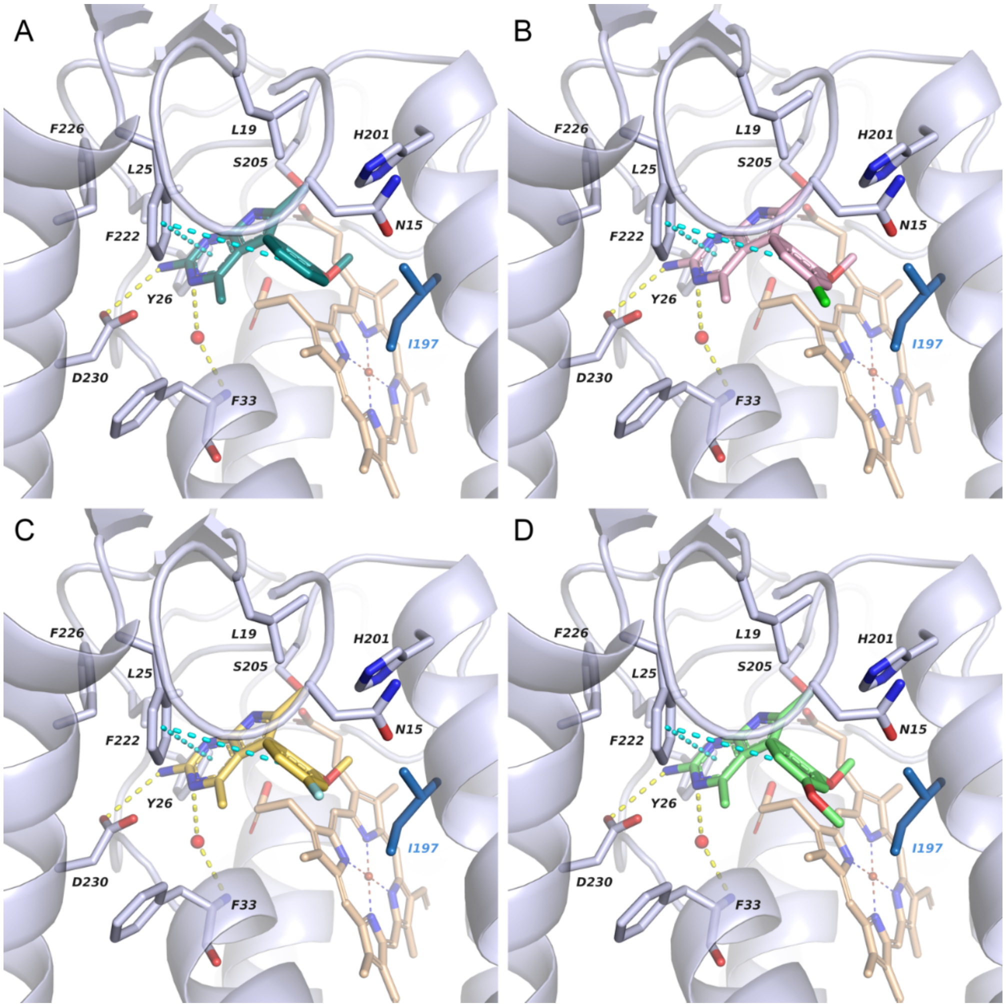

Fig. S15. Predicted binding modes of compounds 1-4 in the *T. cruzi* cytochrome *b* pocket bearing L197I mutation.

(**A**) compound 1 (**B**) compound 3 (**C**) compound 2 (**D**) compound 4. Only the side chains of the surrounding amino acid residues are shown for clarity, and they are displayed as sticks colored in light grey (WT) and blue (L197I mutation); for residue F33, the main chain is also shown. The heme group is depicted as beige sticks, while the oxygen atom of the conserved water molecule as a red sphere. Binding interactions are represented with dashed lines colored in yellow (hydrogen bond) and cyan (π-π stacking).

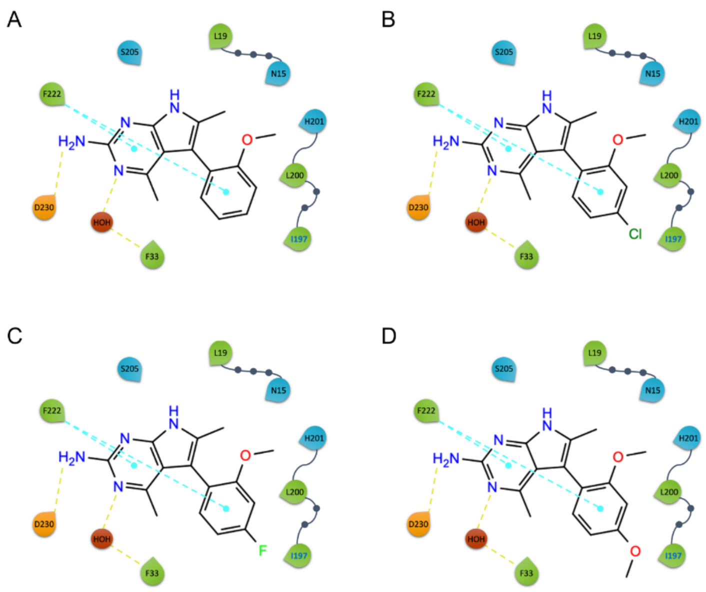

Fig. S16. 2D ligand-protein interaction diagrams of the predicted binding modes of compounds 1-4 in the *T. cruzi* cytochrome *b* pocket bearing L197I mutation. (A) Compound 1. (B) Compound 3. (C) Compound 2. (D) Compound 4. Binding interactions are represented with dashed lines colored in yellow (hydrogen bond) and cyan (π-π stacking)

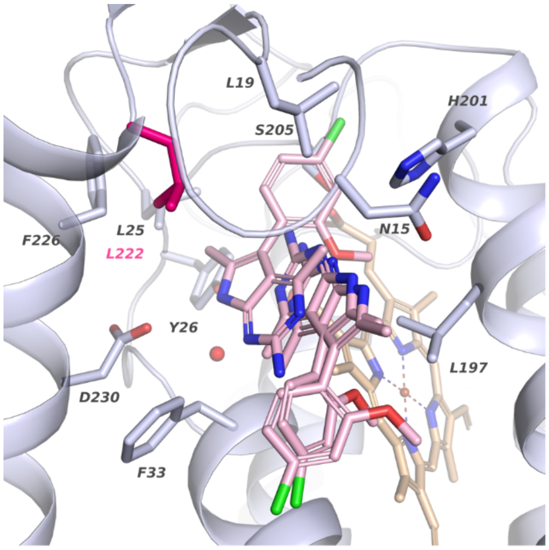

Fig. S17. Predicted binding modes of compound 3 in the *T. cruzi* cytochrome *b* pocket bearing F222L mutation. The first three docking poses are reported, and they are represented as pink sticks. Only the side chains of the surrounding amino acid residues are shown for clarity, and they are displayed as sticks colored in light grey (WT) and magenta (F222L mutation). The heme group is depicted as beige sticks, while the oxygen atom of the conserved water molecule as a red sphere.

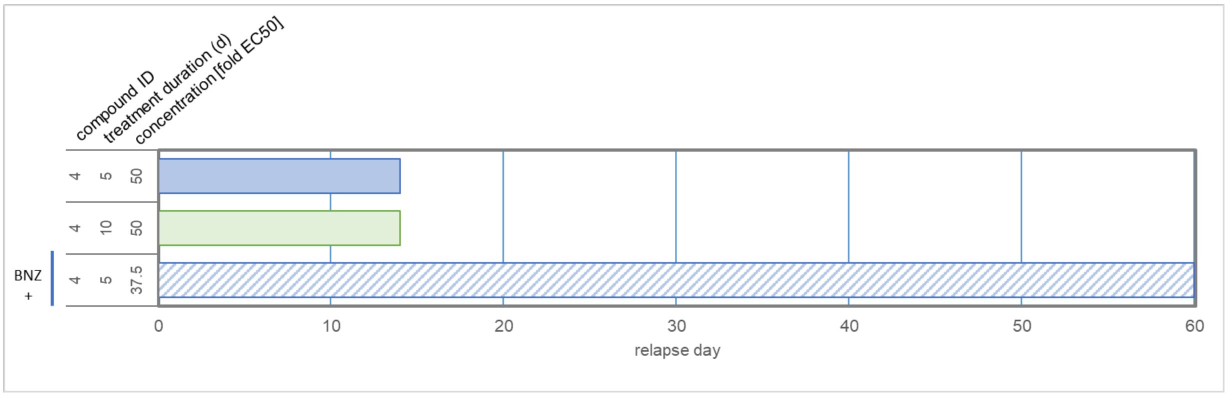

Fig. S18. Washout outgrowth assay for compound 4.

Intracellular parasites were subjected to treatment at the indicated concentrations for either five (blue) or 10 days (green), followed by extensive washout of compound and assessment of parasite relapse for 60 days post washout. Relapse day indicates the first day after washout that parasites were observed microscopically. Combination treatment (hashed bars) used benznidazole at 12.5-fold EC_{50}.

Supplementary tables

Table S1. Efficacy of compounds 2 and 3 against chronic *T. cruzi* infections with different dosing regimens and comparison with BNZ.

Compound	Daily dose (mg/kg)	Treatment length (days)	No. cured/ no. tested
2	50 bid	10	2/6
	100 bid	10	2/4[a]
	50 bid	21	2/6
3	50 bid	10	4/6
	50 bid	20	2/6[b]
BNZ	100 qd	10	3/3
	100 qd	20	6/6

Test compounds are administered at least 80 days post infection with benznidazole and vehicle-only treated groups as controls. Mice were only designated as cured if they were bioluminescence negative by both *in vivo* and *ex vivo* imaging, following immunosuppressive treatment. qd; quaque die (once daily), bid; bis in die (twice daily). [a]Following first day of treatment two mice were culled due to toxicity. Dosing protocol changed to once daily and no further signs of toxicity observed during treatment. [b]Dosing regimen was changed 11 bid + 9 qd due to tolerability issues.

Table S2. Bioanalysis of samples from *in vivo* efficacy studies with compounds 2 and 3 against chronic *T. cruzi* infections with different dosing regimens.

Compound 3 (50mg/kg bid)				
**Days**	**C_{max} Day 1 (ng/ml)**	**C_{max} last day (ng/ml)**	**AUC_{0-7h} Day 1 (h*ng/ml)**	**AUC_{0-7h} last day (h*ng/ml)**
10	325 ± 59	1250 ± 259	1799 ± 243	7793 ± 1564
20	480 ± 69	810 ± 75	2738 ± 388	4917 ± 479

Compound 2 (50mg/kg bid)				
**Days**	**C_{max} Day 1 (ng/ml)**	**C_{max} Day 21 (ng/ml)**	**AUC_{0-7h} Day 1 (h*ng/ml)**	**AUC_{0-7h} Day 21 (h*ng/ml)**
21	870 ± 196	1472 ± 202	5285 ± 1230	8864 ± 1214

Compound 2 (100mg/kg bid)[a]				
**Days**	**C_{max} Day 1 (ng/ml)**	**C_{max} Day 10 (ng/ml)**	**AUC_{0-7h} Day 1 (h*ng/ml)**	**AUC_{0-7h} Day 10 (h*ng/ml)**
10	1252 ± 236	2264 ± 315	6273 ± 1144	11459 ± 1948

[a] Following first day of treatment two mice were culled due to toxicity and dosing changed to qd .

Table S3. Bioanalysis of samples from *in vivo* efficacy studies with compounds 3 co-administered with BNZ for 5 days in a chronic *T. cruzi* mouse infection.

Unbound concentrations of compound 3 (50mg/kg bid)				
**Dosing group**	**C_{max} Day 1 (ng/ml)**	**C_{max} Day 5 (ng/ml)**	**AUC_{0-7h} Day 1 (h*ng/ml)**	**AUC_{0-7h} Day 5 (h*ng/ml)**
3	530 ± 95	1014 ± 185	3024 ± 619	6094 ± 870
3 + BNZ	412 ± 88	977 ± 129	2234 ± 509	5525 ± 663

Unbound concentrations of BNZ (30mg/kg qd)				
**Dosing group**	**C_{max} Day 1 (ng/ml)**	**C_{max} Day 5 (ng/ml)**	**AUC_{0-7h} Day 1 (h*ng/ml)**	**AUC_{0-7h} Day 5 (h*ng/ml)**
BNZ	6928 ± 1302	5230± 1373	18788 ± 3214	12583 ± 1812
BNZ+ 3	5188 ± 1385	3725 ± 1302	15881 ± 4211	11350 ± 4513

Table S4. Whole genome sequencing overview, read counts and coverage.

Cell line	Number of paired reads	Read length	Percentage mapped	Fold coverage	Cytochrome *b* mutation
WT	11970563	150	96.29	32.4	-
Cpd3 RES1	11956517	150	96.16	32.4	Leu197Ile
Cpd3 RES2	11857898	150	94.5	31.5	Phe222Leu
Cpd3 RES3	11981283	150	95.95	32.4	Leu197Ile
Cpd5 RES1	11991442	150	96.04	32.4	Phe222Leu
Cpd5 RES2	11961125	150	96.95	32.6	Phe222Leu
Cpd5 RES3	11890487	150	95.5	32.0	Phe222Leu

Table S5. Bioanalysis of samples from *in vivo* efficacy studies with compounds 4 co-administered with BNZ for 5 days in a chronic *T. cruzi* mouse infection.

Unbound concentrations of 4				
**Dosing group**	**C_{max} Day 1 (ng/ml)**	**C_{max} Day 5 (ng/ml)**	**AUC_{0-7h} Day 1 (h*ng/ml)**	**AUC_{0-7h} Day 5 (h*ng/ml)**
4 (50mg/kg bid)	2934 ± 294	3736 ± 628	16176 ± 776	17378 ± 1605
4 (30 mg/kg bid) + BNZ (30mg/kg qd)	1931 ± 261	1577 ± 161	10441± 1994	6773 ± 311
4 (10 mg/kg bid) + BNZ (30mg/kg qd)	496 ± 82	650 ± 203	2419 ± 453	1488 ± 292

Table S6. Percentage inhibition of human bc1 for multiple replicates at various concentrations of compound 3

Compound 3								
**Concentration µM**	**Data for individual replicates**						**Average % inhibition**	**SD**
	**% inhibition**	**% inhibition**	**% inhibition**	**% inhibition**	**% inhibition**	**% inhibition**		
**200.00**	59.63	22.88	27.88	27.19	-35.32	30.21	22.08	31.08
**66.67**	-178.30	13.25	22.71	-32.39	-2.98	16.87	-26.81	76.83
**22.22**	-14.09	-16.93	21.42	11.31	-5.13	ND	-0.68	16.55
**7.41**	-1.93	0.10	21.95	17.90	0.73	-3.46	5.88	11.05
**2.47**	-19.11	-13.68	7.11	15.66	-8.81	14.23	-0.76	15.00
**0.82**	-55.04	-6.88	19.61	6.94	1.93	10.74	-3.78	26.62
**0.27**	-31.71	-5.91	16.57	7.05	0.38	2.42	-1.87	16.43
**0.09**	-102.79	-0.39	23.45	-1.32	-77.48	6.58	-25.32	51.61

Table S7. Percentage inhibition of human bc1 for multiple replicates at various concentrations of compound 4

Compound 4					
Concentration μM	Data for individual replicates			Average % inhibition	SD
	% inhibition	% inhibition	% inhibition		
200.00	-0.60	5.17	18.62	7.73	9.86
66.67	21.01	18.54	-10.78	9.59	17.69
22.22	11.11	-1.32	9.84	6.55	6.84
7.41	20.02	-3.97	5.85	7.30	12.06
2.47	-7.80	0.71	-1.55	-2.88	4.41
0.82	-0.42	-1.05	-3.03	-1.50	1.37
0.27	-0.73	8.86	-14.09	-1.99	11.52
0.09	-12.21	6.38	ND	-2.92	13.15

Ethical Statements

Mouse Pharmacokinetics and chronic efficacy studies: Animal work was performed under UK Home Office project licenses (PPL 0/8207 and PPL P9AEE04E4) and approved by the London School of Hygiene and Tropical Medicine Animal Welfare and Ethical Review Board. Procedures were performed in accordance with the UK Animals (Scientific Procedures) Act 1986 and the GSK Policy on the Care, Welfare and Treatment of Animals
